# CAN-IMMUNE: A Computational Platform to Facilitate Cancer-Specific Neoantigen Identification

**DOI:** 10.34133/csbj.0274

**Published:** 2026-11-13

**Authors:** Sanjay S.G. Krishna, Mohammad Shahbazy, Kerry A. Mullan, Mohammadreza Dorvash, Gabriel Goncalves, Ritchlynn Aranha, Prithvi Raj Munday, Yuheng Jia, Pouya Faridi, Anthony W. Purcell, Chen Li

**Affiliations:** ^1^ Department of Biochemistry and Molecular Biology, Immunity and Cancer Programs, Biomedicine Discovery Institute, Monash University, Clayton, VIC, Australia.; ^2^ Department of Immunobiology, Yale School of Medicine, New Haven, CT, USA.; ^3^ Monash Proteomics & Metabolomics Platform, Department of Biochemistry and Molecular Biology, Monash Biomedicine Discovery Institute, Monash University, Clayton, VIC, Australia.; ^4^ Centre for Cancer Research, Hudson Institute of Medical Research, Clayton, VIC, Australia.; ^5^ Department of Medicine, School of Clinical Sciences, Monash University, Clayton, VIC, Australia.; ^6^ School of Computer Science and Engineering, Southeast University, Nanjing 210096, Jiangsu, China.

## Abstract

Neoantigens are formed due to the somatic mutations that arise during carcinogenesis; they give rise to unique neo-peptides bearing cancer-specific mutations that are presented on the surface of the tumor in complex with human leukocyte antigen (HLA) molecules for scrutiny by T cells. Some neoepitopes can be recognized by tumoricidal T cells and serve as targets for cancer vaccines and immunotherapies. Liquid chromatography-mass spectrometry identifies human leukocyte antigen-presented peptides through spectral matching against reference proteome databases. However, reference proteome databases usually contain only canonical sequences and lack cancer-specific mutations, limiting neoantigen discovery. To tackle this challenge, we developed a comprehensive platform, CAN-IMMUNE, to browse, search, and generate cancer-specific mutant peptide libraries, thereby enabling the identification of mutant peptides during liquid chromatography-mass spectrometry spectral searches. Major highlights of CAN-IMMUNE include (a) 4,531,600 unique mutant peptides across 55 curated cancer types from 2,052 cancer cell lines and 31 tissues documented to facilitate the generation of bespoke cancer-specific mutant peptide libraries, (b) MutPep, a component of CAN-IMMUNE to allow local generation of mutant peptide libraries using inputs from users, and (c) a computational strategy for prioritizing mutant peptides. We showcased the use of CAN-IMMUNE by searching immunopeptidomics datasets of triple-negative breast cancer and melanoma cell lines. Using CAN-IMMUNE libraries, we computationally identified and prioritized mutant peptides not represented in canonical reference proteome searches, thereby providing potential neoantigen candidates for further immunogenicity assessment. We anticipate that CAN-IMMUNE can accelerate the identification of cancer neoantigens for the development of targeted cancer immunotherapies. CAN-IMMUNE is freely available at https://canelib.erc.monash.edu.

## Introduction

Cancer is a complex disease primarily caused by mutations in genes that regulate cell growth, proliferation, division, and apoptosis [1–3]. While germline mutations play important roles in cancer risk and susceptibility [4], somatic mutations are the major source of cancer neoantigens [5,6]. Neoantigens are a type of antigen that can be formed by various cancer-specific changes and mechanisms, such as somatic mutations, aberrant RNA splicing, protein posttranslational modifications, and the integration of viral open reading frames in virus-induced cancers [6,7]. Abundant immunogenic tumor-specific neoantigens have emerged as promising targets for cancer immunotherapy. In cancer cells, these neoantigens are usually from tumor-specific antigens and are presented to the cell surface via the antigen processing and presentation pathway in complex with human leukocyte antigen (HLA) molecules [8]. Antigen processing and presentation can be further stimulated by interferon-gamma (IFNg), which up-regulates the antigen processing and presentation machinery and has been shown to increase and diversify the peptides presented by HLA molecules [9,10]. These neoantigens are subsequently recognized by T cells triggering immune responses [11]. Accurate identification of tumor-specific neoantigens is therefore of great importance for developing more effective cancer vaccines and immunotherapies.

The characterization of neoantigens has been significantly facilitated by advances in experimental and computational techniques [12,13], including immunogenomic and immunopeptidomic approaches. The immunogenomic approach leverages next-generation sequencing [12] techniques (e.g., whole-genome sequencing) to predict potential neoantigens, followed by computational and experimental assessments of their binding affinity with HLA allomorphs and immunogenicity. To date, numerous statistical approaches including advancements in machine learning algorithms have been developed to predict cancer neoantigens [13], such as Neopepsee [14], MuPeXI [15], pTuneos [16], and NeoFlow [17]. In addition to these neoantigen prediction tools, there are also integrated multiomics tools (e.g., pVACtools [18], iPepGen [19], and NeoDisc [20]) that combine genomic mutation information with HLA-binding prediction and immunogenicity prioritization. In contrast to immunogenomic approaches, immunopeptidomic methods aim to directly identify and quantify HLA-bound peptides, including neoantigens using liquid chromatography-mass spectrometry (LC-MS/MS) techniques [8,21,22]. A typical workflow for immunopeptidome identification usually involves 4 key steps, including sample preparation, purification of major histocompatibility complex-peptide complexes, LC-MS/MS acquisition, and subsequent data analysis and validation [8]. During the LC-MS/MS acquisition step, once the spectral data are acquired, 2 basic search strategies can be employed, including a *de novo* search and a database search. While *de novo* sequencing does not depend on a reference database and can in principle recover mutation-derived sequences, it remains limited in immunopeptidomics due to its higher false-identification rates, the absence of a rigorous target-decoy framework for false discovery rate (FDR) control, sequence ambiguity arising from isobaric and near-isobaric residues (e.g., I/L and K/Q), and strong dependence on the spectral quality and the training data used [23–25]. Recent deep learning-based *de novo* tools have considerably improved peptide sequencing accuracy and coverage [26]. However, data-dependent acquisition followed by database searching against well-curated sequence databases remains the method of choice for high-confidence neoepitope identification [27]. For the database search, a reference proteome library is required for the search engine to enable accurate peptide identification and sequence determination. However, the reference proteome libraries typically only contain canonical wild-type protein/peptide sequences and lack cancer-specific mutation information, hindering the identification of cancer-specific neoantigens. Therefore, mutation information from next-generation sequencing or public databases must be incorporated into the proteome library to identify potential neoantigens effectively [28]. Several databases of mutated peptides and cancer neoantigens have been established in this regard, including XMAn [29], Neodb [30], dbPepNeo2.0 [31], CEDAR [32], and TSNAdb v2.0 [33]. These libraries were constructed using experimentally validated cancer neoantigens and mutation data sourced from public databases and datasets, such as the Catalogue of Somatic Mutations in Cancer (COSMIC) [34]. However, mutant peptide libraries (i.e., mutant peptide sequence databases in FASTA format used as input for immunopeptidomics searching) often lack detailed sample or cell line information, making it challenging to build bespoke libraries for specific cell lines or tissues. Moreover, cancer neoantigen databases may contain a limited number of validated neoantigens for specific cancer types. There is therefore a pressing need for a robust pipeline capable of generating ready-to-use mutant peptide libraries from diverse sources, including public databases and user-provided protein mutation information

In this study, we introduce CAN-IMMUNE, a comprehensive platform to facilitate cancer neoantigen discovery. CAN-IMMUNE comprises 4 core components: (a) an interactive and user friendly interface for mutant peptide search and browse functions, (b) downloadable bespoke mutant peptide libraries with associated metadata, such as transcript-level tissue expression from GTEx [35] of associated genes and on-demand HLA class I binding prediction for each wild-type and mutant peptide, (c) a documented application programming interface (API) providing programmatic access to all queries available through the web interface, and (d) a Python-based package, MutPep, to streamline mutant peptide annotation from various resources, including whole-exome sequencing data in the Mutation Annotation Format (MAF), mutation databases such as COSMIC, or user-provided mutation information. By cross-referencing COSMIC, CCLE (Cancer Cell Line Encyclopedia) [36], mainstream cancer neoantigen databases, and published literature, CAN-IMMUNE harbors 4,531,600 unique mutant peptides across 55 curated cancer types, 2,052 cancer cell lines, 31 tissue lineages, 163 histological sites, and 20,081 genes. Importantly, the mutant peptides catalogued in CAN-IMMUNE are *in silico* generated 25-mer windows centered on annotated cancer missense mutations, allowing the identification of potential mutant peptides with various lengths. Through the interactive interface of CAN-IMMUNE, users can browse, search, and download mutant peptide sequences and their associated metadata for their LC-MS/MS database search. MutPep accepts mutation data in MAF and cross-references RefSeq (RRID:SCR_003496) [37] and UniProt (RRID:SCR_002380) [38] to generate ready-to-use mutant peptide libraries tailored to user-provided constraints. As a case study, we re-searched and analyzed our previously published immunopeptidomic data from triple-negative breast cancer (TNBC) [10] and melanoma [9]. For example, using CAN-IMMUNE libraries, we identified 92 mutant peptides (8mer to 14mer) in the MDA-MB-231 breast cancer (BC) cell line, 68 from interferon gamma treated (IFNg+) and 49 from untreated (IFNg−) cells and 25 detected in both. Among IFNg peptides, 65 (~71%) were predicted binders to at least SD of the 6 HLA class I allomorphs of this cell line. We also developed a computational strategy to evaluate candidate mutant peptides, thereby aiding in the discovery of potential neoantigens by shortlisting mutated peptides that can be further experimentally validated.

## Materials and Methods

### Mutant peptide library generation from curated cancer mutation data

Mutation data were collected from 3 main resources: (a) the COSMIC (v104, GRCh38, released February 2026 and retrieved July 2026; RRID:SCR_002260) with 2 separate exports used, including the main catalogue of patient tumor samples and the Cell Lines Project [34], (b) the CCLE (RRID:SCR_013836) [36], distributed through the Cancer Dependency Map (DepMap, release 26Q1; RRID:SCR_017655), (c) published literature [39], and (d) curated cancer neoantigen databases, including TSNAdb v2.0 [33], the Cancer Epitope Database and Analysis Resource (CEDAR) [32], NEPdb [40], Neodb [30], and dbPepNeo2.0 [31]. We retained the source annotation or each mutation and mutant peptides, enabling the source tracking of each database entry. All contributing resources, their versions and coverage are listed in Tables S1 to S4. Detailed data processing is described in Note S1.

We then built a computational pipeline to verify these mutations by cross-referencing their gene names, amino acid sequences and position information with the NCBI Reference Sequence Database (RefSeq) [37] and UniProt [38]. Transcript accessions were mapped to UniProt by exact sequence match rather than identifier lookup to avoid residue-offset errors that a transcript and its nominal protein entry differ in length. Mutations were retained only if the source-annotated wild-type residue matched the corresponding position in the canonical reference sequence. Entries with transcript version mismatches or inconsistent residue positions were excluded. As a result, 12,770,178 records were retained from the COSMIC tissue samples, 1,229,740 from the COSMIC Cell Lines Project, 670,319 from DepMap/CCLE and 56,873 from published literature and curated neoantigen databases (Tables S3 and S4). The overlaps between the difference sources are shown in Fig. S1. We then harmonized the tissue sample metadata, including sample identifiers, primary tissue site and histological classification, so that cell lines and patient tumor samples are described uniformly. We also examined the cell line information against Cellosaurus (RRID:SCR_013869) [41] and the TRON Cell Line Portal (TCLP) (RRID:SCR_003563) [42] to annotate HLA typing. The primary tissue site and histological classification recorded by each source were mapped to the OncoTree ontology (RRID:SCR_026218) [43]. All classification levels used throughout CAN-IMMUNE are defined in Table S5. Furthermore, to annotate gene and transcript-level expression across normal tissues was drawn from GTEx (RRID:SCR_013042) [35]. With these validated mutations, we extracted peptide sequences of 25 amino acids to cover HLA class I peptides together with their flanking context, including 12 residues upstream and downstream of the mutated amino acid. All searched mutant peptides and metadata can be easily exported from CAN-IMMUNE (Tables S6 and S7 and Note S2).

### Construction of the CAN-IMMUNE platform

The implementation of the CAN-IMMUNE was divided into 2 main parts: the front end (client-side) and the back end (server-side). The back-end server of CAN-IMMUNE was implemented using the Python Flask web framework (RRID:SCR_025723), which handles the server-side logic and necessary data processing steps, was employed to store mutation metadata and mutant peptides in separate tables, facilitating efficient data storage and querying. To make CAN-IMMUNE user-friendly, we designed 3 functional modules on the platform, including Dashboard, Browse, Search, and Statistics, together with a documented Representational State Transfer Application Programming Interface) API (FastAPI) that exposes the same queries programmatically. The Browse module allows users to explore selected mutation sources, such as cell lines, tissues, gene, or tumor histological sites. This module handles requests for information processed by the backend Python logic, which is then rendered to the front end via the Jinja Flask template. The Search module enables users to extract mutant peptides by choosing mutation sources (e.g., cell line, tissue, or a combination of both) and filtering on cancer type, gene, and peptide sequence. A variant recurrence filter can help restrict the searched mutations observed in a minimum number of samples within the selected cancer type. Each result row displays the wild-type peptide alongside its mutant form, and every peptide entry carries a unique CAN-IMMUNE identifier that is displayed on the Browse and Search pages and included in all downloadable outputs to facilitate cross-referencing. All filtered search results from CAN-IMMUNE can be downloaded in various formats, including FASTA and CSV (Comma-Separated Values), to support different MS searching platforms for immunopeptidome/neoantigen discovery. Note that it is possible that a mutant peptide (defined by the variant and the protein) could be from various samples/cell lines in a downloaded file. In this case, the mutant peptides are by default nonredundant, and the information of involved samples/cell lines are summarized in the FASTA header. The Statistics module provides an overview of mutation data, including the total number of available mutations, types of missense mutations, and breakdowns of mutations in different cell lines, tissues, and specific genes. Each mutation page reports the wild-type and mutant peptide sequences, gene, the gene transcript accession number and transcript-level expression across normal tissues from GTEx [35], and the resources in which the mutation has been reported. We also provided an interactive panel for wild-type and mutant HLA class I binding prediction for a user-selected allele and peptide length. The prediction runs on demand with NetMHCpan 4.2 [44], using the curated HLA typing of the selected cell line where available in CAN-IMMUNE (sourced from Cellosaurus and TRON). Otherwise, a default HLA list from the IEDB (RRID:SCR_006604) [45] reference set of 29 common class I alleles is used, which account for 98.98% of HLA-A and HLA-B variation in the global population [46–48]. All graphs are visualized using ECharts [49] and Gunicorn was used to manage client-side server request URL redirection. The front-end interface utilizes AJAX to asynchronously interact with the server-side data. CAN-IMMUNE resides on the Nectar Research Cloud administered and managed by Australian Research Data Commons and Monash eResearch Centre.

### Using CAN-IMMUNE to identify potential neoantigens in BC and melanoma immunopeptidomic data

In this study, we demonstrate the identification of potential neoepitopes using the mutant peptide libraries from CAN-IMMUNE for TNBC [10] and melanoma [9] immunopeptidomic data. In the study by Goncalves et al. [10], immunopeptidomics and RNA sequencing (RNA-seq) were performed on both untreated and IFNg stimulated MDA-MB-231 (RRID:CVCL_0062) cells to identify HLA-I and II-bound peptides. The publicly available LC-MS/MS raw data include 3 replicates of these 2 conditions (IFNg+/−) and only HLA-I immunopeptidomics raw data were searched. Faridi et al. [9] conducted large-scale immunopeptidomic experiments of IFNg-treated patient-derived melanoma cell lines, including LM-MEL-44 (RRID:CVCL_UC49) and LM-MEL-53 (RRID:CVCL_UC56) with 2 replicates each. These BC and melanoma cell lines include IFNg-stimulated and unstimulated conditions to demonstrate that the IFNg stimulation can broaden and diversify the immunopeptidome repertoire [9,10]. For MDA-MB-231, we built 3 mutant peptide libraries using (a) MDA-MB-231 cell line-specific mutations (project = “cell lines”, cell line = “MDA-MB-231”), (b) mutations of all 74 available BC cell lines in CAN-IMMUNE (project = “cell lines”, cancer type = “breast carcinoma”, variant recurrence = “>=2”), and (c) mutations of all BC cell lines + BC samples (project = “All”, cancer type = “breast carcinoma”, variant recurrence = “>=2”). Given that the detailed TNBC subtype annotation for biopsies is limited in source data, we collected all mutant peptides from BC tissues in CAN-IMMUNE and combined them with the libraries based on all BC cell lines. For the second and third libraries, due to the huge number of mutations, we only selected mutations found in at least 2 tissue samples/cell lines (i.e., variant recurrence = “>=2”) and their resulting peptides to build the library. While for LM-MEL-44 and LM-MEL-53, as no cell line-specific mutation data were available, we only built 2 mutant peptide libraries using (a) mutations of all 153 melanoma cell lines (project = “cell lines”, cancer type = “melanoma”, variant recurrence = “>=2”), and (b) mutations of all melanoma cell lines + malignant melanoma skin samples (project= “All”, cancer type = “melanoma”, variant recurrence = “>=2”). The detailed breakdown of these libraries is provided in Table S8 and the sequence overlap between these libraries is shown in Fig. S2. In this study, MSFragger (v4.4.1) was used as the primary search engine via FragPipe (v24.0; RRID:SCR_022864) [50,51]. The parameter configurations for spectra search in MSFragger are provided in Table S9. Several key search parameters for MSFragger included “Digest mode = nonspecific/no-enzyme”, “Precursor mass = 20.0 ppm”, “max variable PTM per peptide = 3” modification types including oxidation (M), deamidation (NQ), and acetylation (N-term) as a variable modification, and restricting the peptide length to 7 to 25 amino acids for HLA class I peptide search. More importantly, it is essential to rescore these identified peptides for better FDR control, due to the inflated search space created using the different libraries. MSFragger has integrated Percolator (3.07.1) [52] and MSBooster (1.4.14) [53] for this purpose. In our analysis, we applied 1% subgroup (class-specific) FDR when using FragPipe (v24), considering the potential unbalanced search space in the reference library. We used the “Hyperscore” from FragPipe “psm.tsv” export to quantify how well a candidate peptides theoretical fragment ions match the observed spectrum from the number and intensity of matched *b* and *y* ions, thereby measuring the quality of peptide-spectrum matches. We also used the MSFragger expectation value (E-value) from the same export, which estimates the number of random matches expected to score at least as well as the observed match. Unlike the Hyperscore, the E-value accounts for the number of candidate peptides compared against each spectrum and therefore reflects the size of the search space (Fig. S10). The binding affinity of identified mutated peptides to HLA class I molecules was predicted using NetMHCpan 4.2 (RRID:SCR_018182) [44] with 8mer to 14mer. Peptides were classified as strong binders if the predicted binding rank (%Rank_EL) <= 0.5, weak binders if %Rank_EL > 0.5 and <= 2, or nonbinders if %Rank_EL > 2.

### Automated mutant peptide library generation

We developed a locally executable Python-based tool, MutPep, as a part of our CAN-IMMUNE platform to leverage multiple sources to generate bespoke mutant peptide libraries. MutPep generates mutant peptide libraries in 4 steps: (a) data source processing, (b) mutation validation and mutant peptide mapping, (c) mutation process statistics, and (d) output generation and analysis. MutPep processes mutation data from various inputs, including Variant Call Format (VCF) files, MAF files, COSMIC datasets, and user-defined mutation lists. Given that MutPep can only run locally, input mutation data, including patient-derived data and the generated libraries, remain on the local computer and are not transmitted to the CAN-IMMUNE server. The current version of MutPep specifically processes missense mutations, as these are most relevant for neoantigen discovery [6]. The RefSeq protein database (GRCh38; release 112) [37] is used as the reference sequence library in the package. The standard mutation description, including the wild-type residue, position, mutant residue (e.g., p.A80P), and the protein transcript ID, is required in the input. Similar to CAN-IMMUNE, when constructing mutant peptides, the length of the mutant peptide was set to 25, with 12 amino acids on each side of the mutation. MutPep also generates a brief report for statistics of the generated mutant peptides, including the most frequent mutant amino acid, the mutant peptide length distribution, and the analysis of valid transcript IDs. MutPep offers 3 export options: (a) FASTA format containing mutant peptide libraries optimized for immunopeptidomics search engines, with headers including the gene symbol, transcript accession, amino acid substitution, and sample of origin (Note S1), (b) metadata files containing the user-provided input mappings and transcript IDs for background reference, and (c) HTML reports providing an overview of mutation processing statistics and interactive mutant peptide data tables. Parallel processing techniques using Python (RRID:SCR_008394) built-in multiprocessing function have also been deployed in the package by dividing files into chunks and processing them simultaneously across multiple CPU cores to allow the handling of large mutation datasets.

### Computational evaluation of mutant peptides for prioritizing discovery of potential cancer neoepitopes

We developed a comprehensive approach to computationally evaluate mutant peptides for cancer neoantigen discovery. First, we assessed the peptide–HLA binding affinity via the “%Rank_EL” score from NetMHCpan 4.2 [44] with peptides of 8mer to 14mer. Similarly, peptides with “%Rank_EL”<=0.5, “%Rank_EL”>0.5 and <=2, and “%Rank_EL”>2, are regarded as strong binders, weak binders, and nonbinders, respectively. Second, to assess whether a mutation alters HLA presentation relative to its wild-type form, we calculated the differential agretopicity index (DAI) [54–56] using the log-transformed %Rank_EL value from NetMHCpan 4.2 for each peptide as:

$${\mathrm{DAI}}_{\log_{2}}=\log_{2}\left(\frac{\%\text{Rank}\_{\mathrm{EL}}^{\mathrm{WT}}}{\%\text{Rank}\_{\mathrm{EL}}^{\mathrm{Mut}}}\right)$$ (1)

where positive values indicate that the mutant form is predicted to bind the HLA molecule more favorably than the wild type. Third, we extracted the “Percolator” score generated by MSFragger during the peptide rescoring process. Percolator (v3.7.1; RRID:SCR_005040) analyses peptide-spectrum matches using a statistical mixture modeling approach to discriminate between correct and incorrect identifications. Each peptide identification was assigned a probability score (ranging from 0 to 1) to represent the likelihood of the match being correct based on search engine scores and other features such as mass accuracy and peptide properties. Peptides with Percolator probability score > 0.9 are considered highly confident [52]. Fourth, we extracted the Hyperscore from MSFragger, which was defined above as a measure of spectral match quality. Each of the 4 scores was min–max scaled from 0 to 25 and the combined score (up to 100) was used to rank the peptide. The higher the combined score is, the higher priority the candidate has. Considering NetMHCpan only reports a single predicted binding rank and does not indicate the interaction interface among the peptide and HLA molecule, we then performed peptide–HLA structural analysis using AlphaFold 3 (RRID:SCR_025885) [57]. Default parameters were used and 5 models were constructed for each peptide–HLA complex. This allowed us to explore if a mutation in the HLA binding groove is located at a solvent-exposed position that is available for T-cell receptor contact [58,59]. Since such a mutation at a solvent-exposed position alters the surface presented to the T-cell receptor directly, whereas a mutation at an anchor residue is expected to act on peptide presentation [60]. We recorded and averaged the AlphaFold-derived predicted local distance difference test (pLDDT) score (0 to 100) values [61] as a measure of model confidence and the mean anchor distance values defined as the minimum heavy-atom distance between the mutated residue side chain and the HLA α-chain residues lining the corresponding binding pocket, across the 5 models. These measures were used to interpret the interaction interface among the mutation and the HLA molecule, providing structural insights into potential TCR accessibility [58,62]. Finally, we compared selected mutant peptide spectra (original) to synthetic spectra, where available, or to spectra predicted via Prosit [63], using the Pearson correlation coefficient (PCC). The outputs of the above analyses are provided in the final output file for users to rank the peptides based on their focus. A Jupyter Notebook script has been provided on our GitHub repository to facilitate the above analyses for mutant peptide ranking (URL: https://github.com/sanjaysgk/CanImmune/tree/data-analysis).

## Results

### Using CAN-IMMUNE to generate bespoke mutant peptide libraries

Our CAN-IMMUNE platform implements a comprehensive and integrated approach for generating bespoke cancer-specific mutant peptide libraries. As illustrated in Fig. 1A and B, CAN-IMMUNE comprises 4 interconnected components: mutation data collection, mutation information processing, mutant peptide generation, and data storage/retrieval systems (“Materials and Methods”). In the first component, the mutation data was further cross-referenced with RefSeq [37] and UniProt [38] to ensure annotation accuracy. The platform processes mutation information at both DNA and protein levels, with a particular focus on nonsynonymous mutations in genomic data that produce amino acid changes that could generate neoepitopes. The current version of CAN-IMMUNE encompasses 55 curated cancer types across 2,052 cell lines and 49,611 patient tumor samples, corresponding to 6,812,413 unique mutation variants, spanning 31 OncoTree tissue lineages and 163 histological classifications (Fig. 1C and Table S2). Based on these mutations, we extracted 4,531,600 unique mutant peptides derived from 20,081 genes. These peptides were computationally extracted using a 25-mer sequence window centered on each annotated missense mutation, thereby facilitating immunopeptidomic search. Mutant peptides that have been experimentally identified as cancer neoantigens (i.e., by Neodb, dbPepNeo2.0, NEPdb and/or CEDAR) are flagged as such in the metadata. Among these single nucleotide variants (SNVs), the G➔A mutation was the most frequent (27.7%), followed by the C➔T mutation (21.8%; Fig. 1D). While at the protein level, glutamic acid to lysine (E➔K; 4.7%) was the most prevalent amino acid substitution, followed by arginine to glutamine (R➔Q; 3.3%) and arginine to histidine (R➔H; 3.2%) substitutions (Fig. 1E). The distribution of mutations across tissues lineages (Fig. 1F) demonstrates that among tumor tissue samples, skin, bowel, and lung showed particularly high mutation frequencies (17.5%, 13.9% and 11.4%), consistent with their known high mutational burdens [64]. Importantly, CAN-IMMUNE maintains detailed sample information, such as cancer types, cell lines, and individual tissues/samples, allowing users to select the most appropriate mutation data to build mutant peptide libraries for their specific research context. These mutant peptides can be readily used as reference libraries in standard LC-MS/MS-based immunopeptidomic workflows to identify potential neoantigens that could be missed using canonical protein databases alone.

**Fig. 1.**
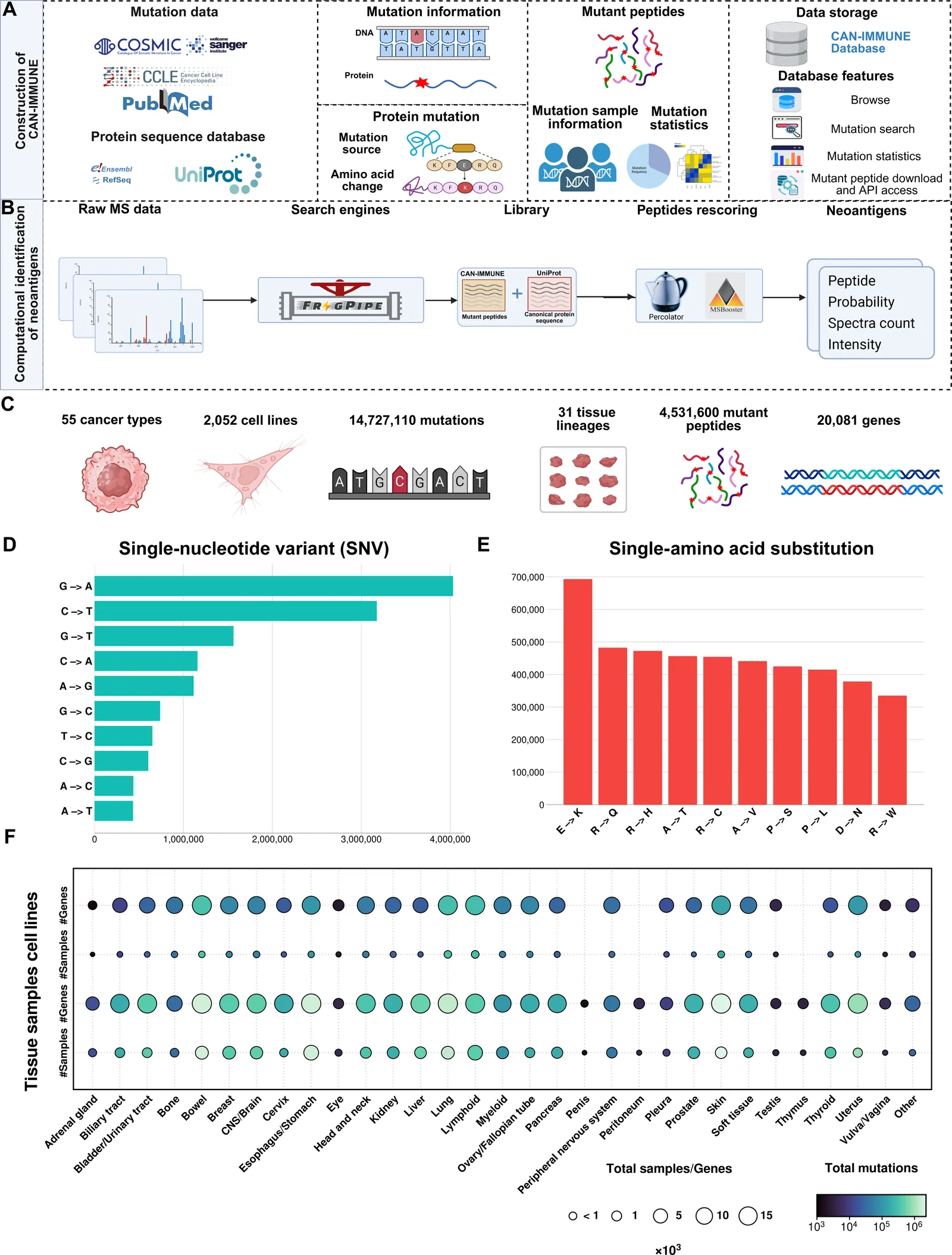
An overview of the CAN-IMMUNE framework and the database statistics. (A) The workflow of constructing cancer-specific mutant peptide libraries in CAN-IMMUNE. (B) The workflow of computational identification of potential neoantigens using the search engine FragPipe. The results discussed in the paper are based on searches by FragPipe. (C) General statistics of CAN-IMMUNE, including the numbers of cancer types, cell lines, mutations, mutant peptides, and genes documented in the database. (D) Top single nucleotide variant and (E) single amino acid substitutions in CAN-IMMUNE across tissues and cell lines. (F) Mutation counts across the 31 tissue lineages in CAN-IMMUNE, plotted alongside sample numbers (cell lines + tissue biopsies). Tissue sites refer to the source-annotated 42 anatomical sites, which were mapped to the 31 OncoTree tissue lineages. Multiple tissue sites can map to the same cancer type. Values were calculated using a base-10 logarithm (Log10). The underlying data are tabulated in Table S2.

### CAN-IMMUNE database interface and functionality

The web portal of CAN-IMMUNE provides 4 interconnected and user-friendly interfaces with a documented REST API (Fig. 2), including Dashboard, Browse, Search, and Statistics, enabling comprehensive and bespoke generation of cancer-specific mutant peptide libraries. The “Dashboard” interface provides an overview of the database resource with key statistics of the current version, including the total number of mutant peptides, cell lines, and cancer types. We also designed and implemented various visual representations to demonstrate data distribution across tissue types and cancer histology categories, offering users immediate insights/statistics into the scope and content of CAN-IMMUNE (Fig. 2A). The “Browse” page lists cell lines, tissue samples, cancer histological sites, and genes with detailed metadata. Users can navigate through the structured lists to locate samples of interest (Fig. 2B). Each entry on the lists leads to a webpage containing comprehensive information, including mutation counts, sample information, mutation source, and a full list of wild-type and mutant peptides (Fig. 2C). Users can explore mutation patterns via these interactive charts that visualize mutation distributions in tissue types and cell lines, enabling identification of the same mutations appearing in various cell lines/tissues. Each peptide entry contains detailed metadata including gene source, mutation type, amino acid change, and flanking sequences (Fig. 2D). The “Search” page provides an intuitive query system for users to extract mutant peptides by keywords including gene, mutant or wild type peptide, cell line, tissue, cancer type, variant recurrence, and HLA typing where available (Fig. 2E). The search results are presented in a tabular format with pagination support, providing detailed peptide information (Fig. 2E). All search results can be downloaded in the FASTA format with accompanying metadata tables in the CSV format for direct use in proteomics and immunopeptidomics platforms such as FragPipe (v24.0) [50], DIA-NN [65], and PEAKS [66].

**Fig. 2.**
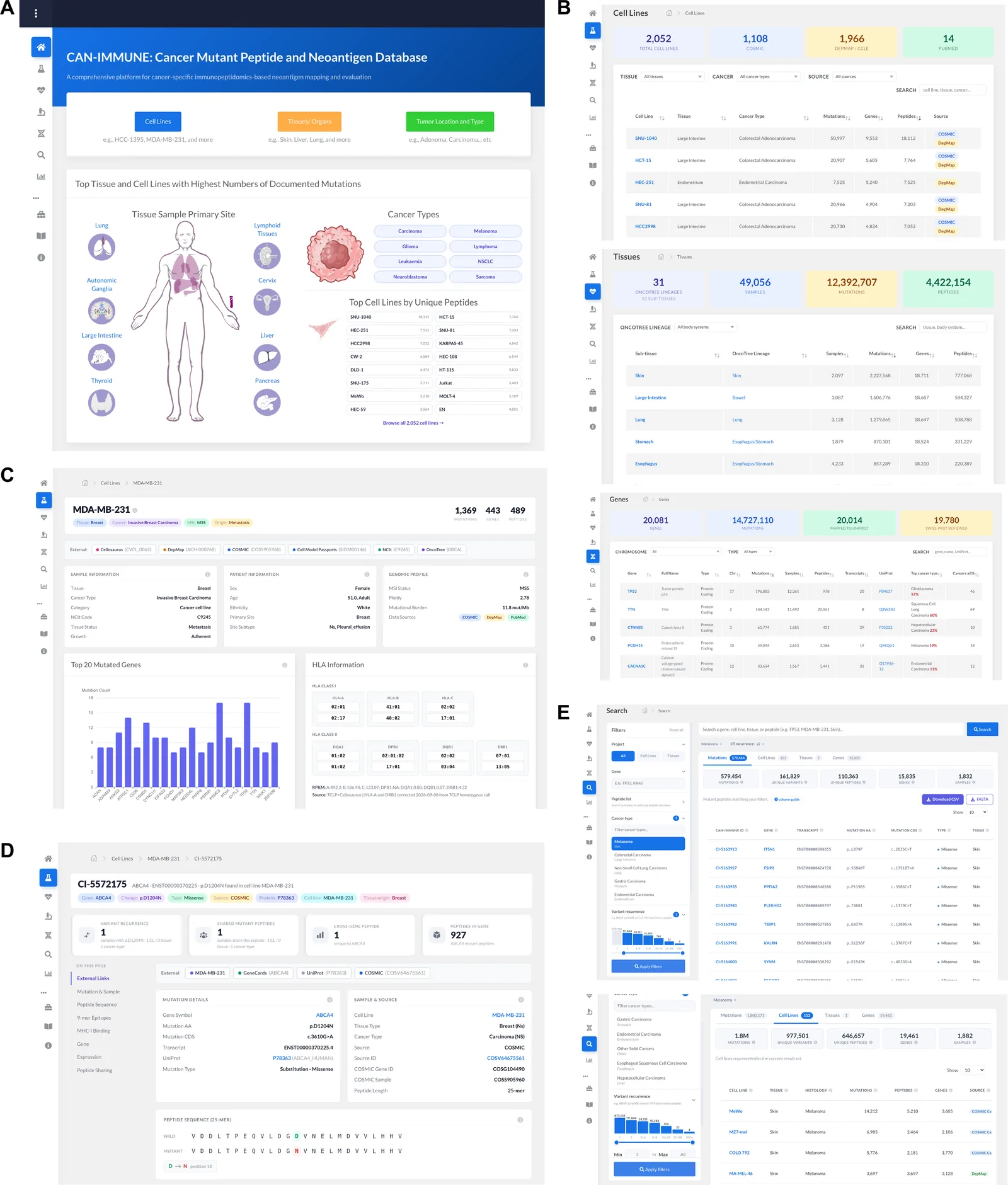
An overview of the CAN-IMMUNE platform. (A) The homepage of CAN-IMMUNE showing tissue systems biology map and cancer primary histology classification with a dashboard displaying real-time statistics of the database including the numbers of data from different data source (Catalogue of Somatic Mutations in Cancer [COSMIC], Cancer Cell Line Encyclopedia [CCLE], and literature). (B) An interactive cell line browser interface enabling exploration of cancer cell lines with filterable keywords including tissue type and mutation status. The tissue sample list below provides categorized access to tissue samples with related biological annotations. (C) A mutant peptide lists showing detailed mutation information. The interface includes visualization of mutation frequency across selected cell lines. (D) Sample-specific mutation details with validation status indicators (middle panel) and comprehensive information for specific mutant peptides including sequence, structural distribution, and tissue prevalence. (E) The database query interface with advanced search functionality. The search form allows filtering by sequence, tissue/cell line, cancer type, variant recurrence, etc. Search results are displayed in a tabular format with comprehensive mutation information including cell line origin, transcript accession, mutation details, primary tissue, and full peptide sequences.

### MutPep: A key toolkit of CAN-IMMUNE for generating mutant peptide libraries

Despite the comprehensive mutant peptide library available in CAN-IMMUNE, it may not capture rare or sample-specific mutations absent from public databases. To address this, we developed MutPep, a standalone Python-GUI tool as part of CAN-IMMUNE that enables users to generate bespoke mutant peptide libraries from their own mutation data, compatible with various mass spectrometry search engines (Fig. 3A). To demonstrate the MutPep functionality, we performed a reproducible example below. We first downloaded a MAF file (https://portal.gdc.cancer.gov; downloaded on 21/03/2025) for BC whole-exome sequencing from The Cancer Genome Atlas Program (TCGA) GDC data portal [67]. We uploaded this dataset (5,064 mutations) to MutPep (Fig. 3B) using GRCh38 as the reference sequence database with 12 flanking amino acids on each side of the mutation position (total length: 25 aa). Of these, 1,609 entries were excluded as nonmissense mutations, leaving 3,455 missense mutations for processing. MutPep successfully matched ~93.9% (*n*=3,245) to their source transcript IDs with verified amino acid changes, while 210 transcript IDs could not be mapped due to version discrepancies between the input data and the reference database. The results panel of MutPep demonstrates top mutations in the provided datasets, such as lysine (K), glutamine (Q), and asparagine (N) substitutions (Fig. 3B). Leveraging multithreading capabilities, MutPep processed the entire 3,455 mutations in approximately 1 min on a PC equipped with standard computing hardware (8-core CPU and 16GB RAM), demonstrating significant performance optimization for high-throughput mutation analysis workflows.

**Fig. 3.**
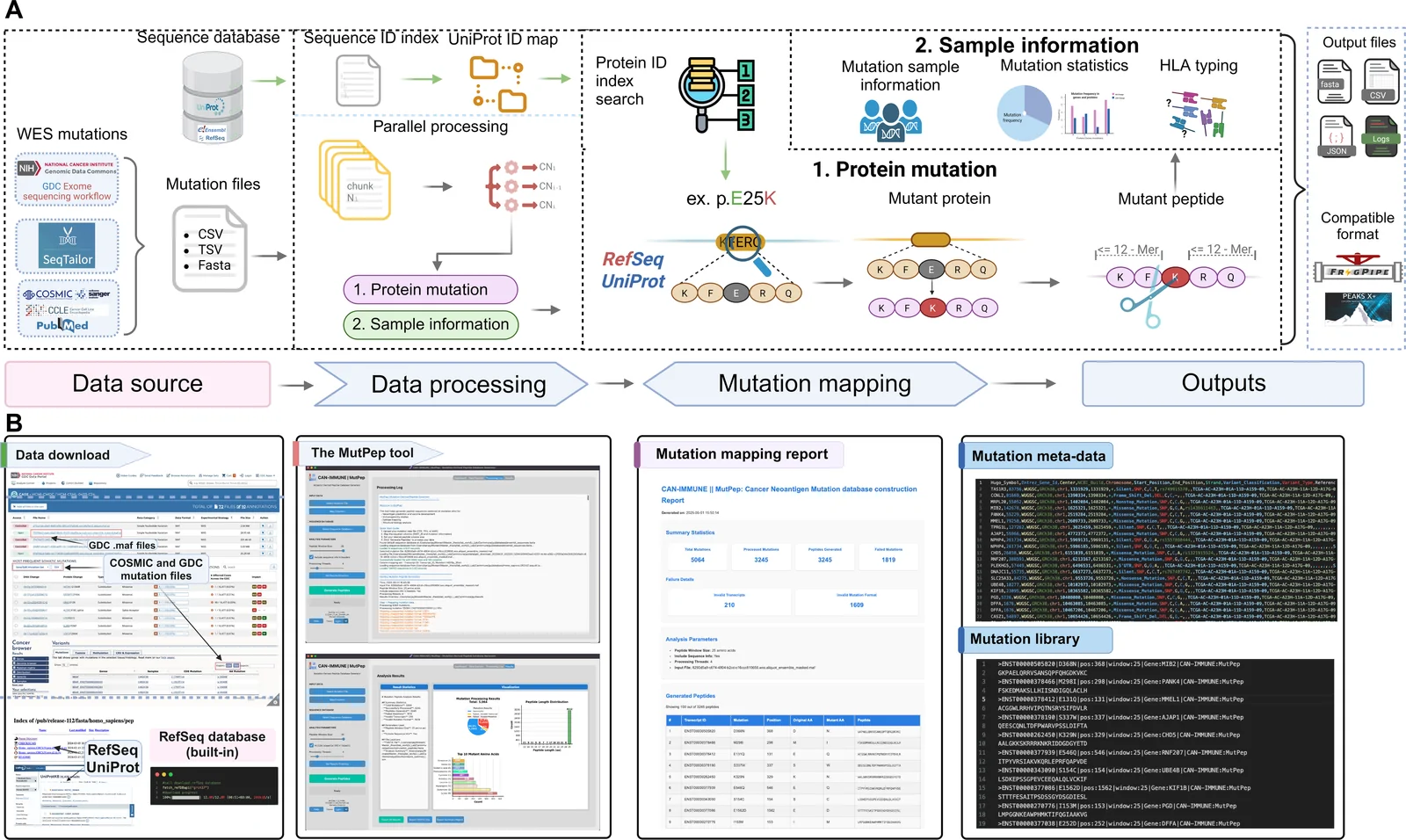
Using the MutPep program to generate mutant peptide libraries using user-provided mutation data. (A) The workflow of the MutPep program to generate searching engine compatible mutant peptide libraries. (B) An example showing the process of using breast cancer whole-exome sequencing [67] data for generating multiple outputs, including statistical analysis, mutation mapping reports, mutation metadata, and the mutant peptide library.

### Identification of potential neoantigens for BC and melanoma using CAN-IMMUNE

To validate the utility of the CAN-IMMUNE database for neoantigen discovery, we generated mutant peptide libraries to interrogate immunopeptidomics datasets from TNBC (MDA-MB-231) [10] and melanoma [9] cell lines (LM-MEL-44 and LM-MEL-53) using CAN-IMMUNE. We then performed library-based searches and analyses of immunopeptidomic data for cell lines using reference proteomes augmented with the cancer specific CAN-IMMUNE libraries. Thus, we re-searched our previously published LC-MS/MS data of TNBC and 2 melanoma cell lines using FragPipe with a standard 1% FDR threshold applied at both the global and subgroup (class-specific) level, followed by peptide rescoring using Percolator [52] and MSBooster [53] and a peptide–HLA binding analysis using NetMHCpan 4.2 [44]. We identified a number of mutant peptides for BC and melanoma using the peptide libraries extracted from CAN-IMMUNE (Fig. 4). Specifically, using the MDA-MB-231 specific library from CAN-IMMUNE, we identified 8 unique mutant peptides (2 shared by IFNg+ and IFNg−, 5 by IFNg+ only and 1 by IFNg- only) (Table S10). Generally, regardless of the type of mutant peptide libraries from CAN-IMMUNE, MSFragger identified more mutant peptides from IFNg-treated cell lines, consistent with the finding that IFNg induction can broaden and diversify the immunopeptidomic repertoire [9,10] (Fig. 4A). In addition, with the increasing size of the mutant peptide library, more mutant peptides were identified (Fig. 4A and Figs. S3 and S4), suggesting that the integration of cell line databases with broader tissue-specific datasets substantially increased the number of identifiable mutant peptides. For example, using the MDA-MB-231-specific mutant peptide library, we found 10 mutant peptides (8 unique) across the IFNg+ and IFNg− conditions (Fig. S3A), while these numbers increased to 99 (76 unique mutant peptides) when using the extended mutant peptide library using all breast carcinoma cell lines and BC tissues (Fig. S3A). We demonstrated the numbers of peptides that have been rescored using Percolator [52] and MSBooster [53] embedded in FragPipe. The distribution of rescoring probabilities for the mutant peptides identified in MDA-MB-231 is shown in Fig. S5. Peptide length distributions and HLA class I binding motifs for each search database are shown in Figs. S6 to S8 for MDA-MB-231, LM-MEL-44, and LM-MEL-53, respectively. NetMHCpan 4.2 was then used to predict HLA-binding for these mutated peptides across different cell lines in IFNg+ and IFNg− conditions (Fig. 4B). Across all BC mutant peptide libraries, approximately 71% unique mutated peptides (65 of 92) were predicted to bind at least 1 sample-relevant HLA allotype, with the distribution of predicted %Rank_EL across the 6 MDA-MB-231 allotypes shown in Fig. S9. Specifically, using the MDA-MB-231-specific mutant peptide library, we identified 8 unique mutant peptides across the IFNg-treated and untreated conditions, all were predicted as strong binders to at least 1 HLA-allotype expressed by this cell line. When we expanded our search using the mutations of all BC cell lines + BC tissues, we observed 75% (57 of 76) unique mutant peptides binding at least 1 of the 6 MDA-MB-231 HLA allotypes, presenting an approximately 7-fold increase in identified binders (8 to 57 binders) compared to the MDA-MB-231-specific library. Similarly, for melanoma cell lines, 66% (42 of 64) unique mutant peptides in LM-MEL-44 and 52% (29 of 56) in LM-MEL-53 were predicted to bind to at least 1 relevant HLA allotype. Specifically, using the mutant peptide library of all melanoma cell lines, we identified the highest number of strong binders (3 peptides) to HLA-B*40:01 and the highest number of weak binders (4 peptides) to HLA-B*44:02 in IFNg-treated LM-MEL-44 cells compared to other allotypes. While using the mutation library of all melanoma cell lines + malignant skin tissues, we identified the highest number (18 peptides) of HLA-A*02:01 binders in LM-MEL-44 IFNg+ cells.

**Fig. 4.**
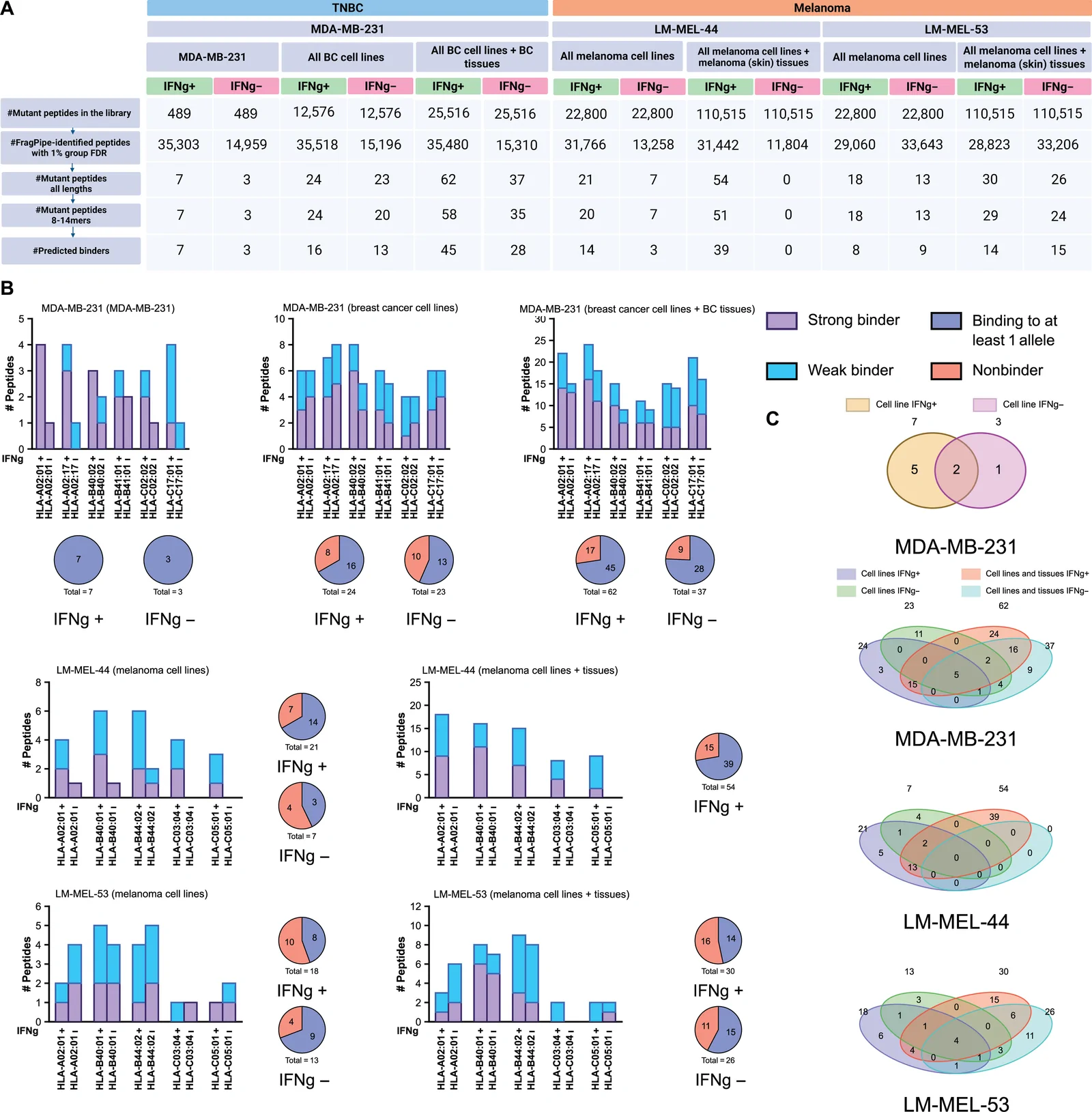
Identification of mutant peptides from breast cancer and melanoma cancer cell lines using CAN-IMMUNE at 1% false discovery rate (FDR). (A) The detailed results demonstrating the identification of mutant peptides and human leukocyte antigen (HLA) binders for breast cancer and melanoma cell lines (IFNg+/−). (B) Breakdowns of NetMHCpan-predicted strong, weak, and nonbinders of identified mutant peptides (1% grouped FDR; 8mer to 14mer) for breast cancer and melanoma. Bar charts show the distribution of strong binders (purple), weak binders (blue), across different HLA alleles in breast cancer (MDA-MB-231) and melanoma (LM-MEL-44 and LM-MEL-53) cell lines with and without interferon-gamma (IFNg) stimulation. Pie charts display the total number of peptides identified in each condition and the proportion of binders (strong and weak binders) *vs.* nonbinders. (C) Venn diagrams illustrate overlap of identified mutant peptides using cell line-specific, all cell lines, and all cell lines + tissue mutant peptide libraries.

To understand how library composition influenced mutant peptides identification, we examined the overlap of the identified mutant peptides in IFNg-treated cells using cell line-based libraries and cell lines + tissues libraries (Fig. 4C). For example, we identified 20 (of 24 with all BC cell lines and 62 with all BC cell lines + BC tissues) and 15 (of 21 and 54) overlapping peptides in MDA-MB-231 and LM-MEL-44 IFNg+ cells, respectively and 9 (of 18 and 30) in LM-MEL-53. We also noticed that not all mutant peptides identified using the library of the specific cell line were detected when using the library of all cell lines. Similarly, not all mutant peptides identified using the library of all cell lines can be found when using the library combining all cell lines and tissues (Fig. 4C). This is because these peptides failed FDR-rescoring against the expanded decoy space (Fig. S10). For example, in untreated (IFNg−) LM-MEL-44 cells, 7 mutant peptides identified using the melanoma cell line library were not found when using the library of all melanoma cell lines + skin tissues, likely due to the small number of peptide-spectrum matches for mutant-peptides available for subgroup FDR estimation. The overlap therefore characterizes the sensitivity-specificity trade-off introduced by library size, an important consideration for users to choose when using CAN-IMMUNE to generate mutant peptide libraries. Upon examination, we found these peptides failed the FDR assessment after peptide rescoring when the extended mutant peptide library was used (i.e., all cell lines and tissues). Increasing database size can introduce alternative peptide assignments, as a given spectrum may match either a posttranslationally modified peptide or a peptide with variants (Figs. S3 and S4). This factor therefore should be carefully considered when expanding search databases in immunopeptidomics analysis. The overlaps of identified peptides and mutant peptides across all searches for MDA-MB-231 and the melanoma cell lines are shown in Figs. S11 and S12, respectively.

### Computational evaluation of mutant peptides for neoepitope discovery

Given the large number of mutant peptides identified, experimental validation of all candidates is not feasible. We therefore applied a computational strategy to evaluate candidates before experimental follow-up. In this section, we showcased this strategy with 62 mutant peptides identified in MDA-MB-231 IFNg+ cells using the mutant peptide library of all BC cell lines + BC tissues, of which 58 mutant peptides were 8mer to 14mer and were used in the evaluation. We demonstrate 4 scores (%Rank_EL, DAI, Percolator probability and Hyperscore) of top 10 and last 5 mutant peptides in Fig. 5A. As Percolator [52] is part of FragPipe for peptide rescoring, we selected peptides with a probability score greater than 0.90. All 58 peptides were identified at 1% FDR with a rescoring probability above 0.98. Both rescoring probability and Hyperscore from MSFragger were comparable across the 3 predicted binding classes (median Hyperscore 22.3, 20.3, and 21.3 for strong binders, weak binders, and nonbinders, respectively), indicating that identification quality is independent of predicted binding (Fig. 5B). Note that if PEAKS is used as the search engine, other peptide rescoring approaches can be considered, such as MS^2^Rescore [68]. We then applied NetMHCpan 4.2 to predict peptide–HLA binding for all relevant HLA-allotypes, resulting in 31 (53.4%) strong binders (%Rank_EL <= 0.5) (Fig. 5B and Fig. S9). If a peptide was a strong binder to multiple HLA allotypes, the allele with the lowest %Rank_EL was chosen as the presenting allotype HLA structural analysis (Fig. 5B and Fig. S9). We then selected 2 mutant peptides for a structural study. The first mutant peptide was AMAKIQAK**V** (wild type: AMAKIQAK**I**; P9) from the reticulon-4 protein (RTN4; UniProt accession: Q9NQC3; mutation: I1184V), ranked the first with a combined score of 77 (Fig. 5A). This mutant peptide was independently predicted as an HLA-A binding neoepitope in this cell line and was also confirmed to bind HLA-A2 *in vitro* [69] (Fig. 5C). The second mutant peptide was R**Q**TPSFGVYF (wild type: R**E**TPSFGVYF; P2) from the mitochondrial basic amino acids transporter protein (SLC25A29; UniProt accession: Q8N8R3; mutation: E161Q), the mutant peptide was predicted as a weak binder (Fig. 5D). The spectra comparison between AMAKIQAK**V** (original) and AMAKIQAK**V** (synthetic; Mimotopes, Melbourne, Australia) resulted in a PCC of 0.99 (Fig. 5E), with the corresponding comparison against the Prosit-predicted spectrum shown in Fig. S13. While the PCC for spectra between the mutant peptide R**Q**TPSFGVYF (original) and R**Q**TPSFGVYF (predicted by Prosit (RRID:SCR_026974) [63]) was 0.32 (Fig. 5F). The high PCC for AMAKIQAK**V** supports its identification, whereas the lower PCC of R**Q**TPSFGVYF (0.32) was due to deamidation modification at P2 (Gln), where the Prosit model did not support to predict modification spectra matching *y* and *b* ions (Fig. 5F). Compared with wild-type peptide (AMAKIQAK**I**), the mutant peptide–HLA structural model using AMAKIQAK**V** placed the valine slightly further from the HLA α-chain, with a mean distance of 3.50 ± 0.02 Å against 3.24 ± 0.07 Å across 5 models. NetMHCpan predicted the mutant peptide to be the stronger binder, suggesting that a mutation at an anchor residue can change how the peptide binds without changing the surface of the peptide exposed to T cells (Fig. 5C). In contrast, the models of R**Q**TPSFGVYF showed lower confidence for both mutant and wild-type form pLDDT 87 ± 0.7 and 80 ± 0.6 for R**Q**TPSFGVYF and R**E**TPSFGVYF against 91 ± 0.3 and 89 ± 0.5 for AMAKIQAK**V** and AMAKIQAK**I**, respectively. The mean anchor distances were similar between the 2 forms (3.35 ± 0.01 Å against 3.36 ± 0.02 Å). However, R**Q**TPSFGVYF demonstrated highly variable pLDDT across 5 models, suggesting a less well-defined peptide–HLA complex (Fig. 5D). On the other hand, AMAKIQAK**V** shows consistent pLDDT across all 5 predicted models (peptide–HLA pLDDT score 91 ± 0.3, mean ± SD across the 5 models) (Fig. 5C). This demonstrates that our evaluation strategy can help understand 4 different scores (%Rank_EL, DAI, Percolator probability, and Hyperscore) and highlight the candidates worth experimental validation. Note that these analyses require further experimental validation, such as binding affinity measurements with synthetic peptides, before any candidates are selected.

**Fig. 5.**
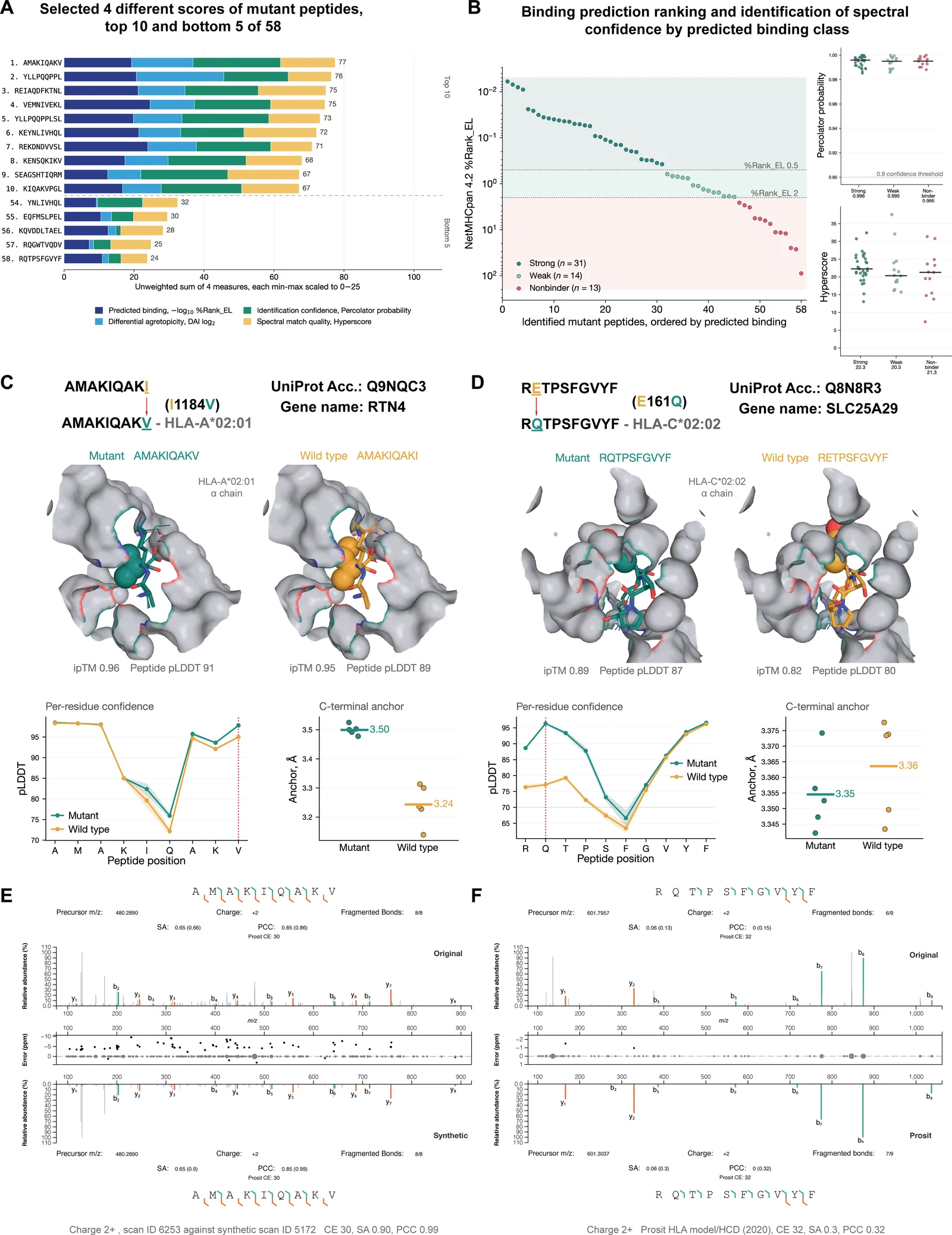
A computational strategy to validate the identified 58 mutant peptides (8mer to 14mer) for neoantigen discovery. (A) Top 10 and bottom 5 peptides based on their combined scores. (B) Mutant peptide ranking using NetMHCpan 4.2 and the peptide identification confidence based on peptide rescoring probability and their Hyperscore by predicted binding class. (C) Peptide-human leukocyte antigen (HLA) structural analysis of AMAKIQAKV (RTN4; UniProt: Q9NQC3), showing the AlphaFold 3 modeled peptide–HLA complex of the mutant and its wild-type counterpart AMAKIQAKI on HLA-A*02:01. The per-residue confidence and the C-terminal anchor distance across 5 models are shown. (D) Peptide–HLA structural analysis of RQTPSFGVYF (SLC25A29; UniProt: Q8N8R3). (E and F) The mirror plot illustrating the spectra comparison between the original peptide and its synthetic peptide for AMAKIQAKV (E) and predicted spectra of RQTPSFGVYF (F) by Prosit, respectively.

## Discussion

The discovery of cancer neoantigens plays a critical role in developing effective cancer vaccines and immunotherapies [7]. In recent years, LC-MS/MS followed by a database search has emerged as a leading technique for identifying and quantifying neoantigens, as it enables direct profiling of potentially antigenic peptides presented on the surface of tumor cells. However, the use of canonical proteome libraries in the database searches hinders the characterization of mutant peptides, as they lack sample- and tumor-specific mutation information. Several computational frameworks have been developed to facilitate neoantigen discovery using genomic and transcriptomic data, such as the pVAC tools [18], iPepGen [19], and NeoDisc [20]. Although these approaches provide powerful frameworks for neoantigen discovery, they heavily rely on sample-specific sequencing data with additional costs, such as RNA-seq or whole-exome sequencing, to generate customized proteogenomic search databases. In contrast, CAN-IMMUNE provides customizable search, retrieval, and extraction of mutant peptide libraries for searching raw LC-MS/MS spectra data without the requirement of RNA-seq or whole-exome sequencing. XMAn v2 [29] is a similar online resource to CAN-IMMUNE, which contains 2,539,031 mutated peptide entries but is a static FASTA file last updated in 2018 with no sample-level metadata. On the other hand, current neoantigen-specific databases have considerably smaller datasets. For example, dbPepNeo2.0 [31], TSNAdb v2.0 [33], and Neodb [30] contain 801, 1,856 (access date: 2026 Mar 19), and 1,463 high-confidence experimentally validated neoantigens, respectively. However, as demonstrated in our functional comparison between CAN-IMMUNE and these resources (Table S11), none of these databases provides ready-to-use mutant peptide libraries linked to specific cell lines or tissues for LC-MS/MS database searching. Our user-friendly CAN-IMMUNE addresses this gap directly, providing 4,531,600 unique mutant peptides derived from 6,812,413 unique missense variants covering 2,052 cell lines and 55 curated cancer types, with detailed cell line and tissue metadata, enabling the construction of bespoke, context-specific libraries that are not achievable with existing resources. The MutPep tool can generate custom libraries from user-provided MAF or VCF input within approximately 1 min on standard hardware. The key to the construction of CAN-IMMUNE is the curation of high-quality cancer-associated mutation data from literature and high-quality cancer mutation databases, such as COSMIC, which, for example, assisted with neoantigen discovery for hepatocellular carcinoma using HepG2 cells [28].

We demonstrated the practical use of different types of mutant peptide libraries on our BC and melanoma datasets. We found that generally larger libraries led to an increasing number of mutant peptides identified. For example, in MDA-MB-231 (combined IFNg +/−) we identified 10 mutant peptides (8 unique) using the cell line-specific library, rising to 99 (76 unique) with the library of all breast carcinoma cell lines combined with BC tissues. Given the large size of the libraries used, we used a stringent group-FDR of 1% as the primary filter, to avoid the potential FDR inflation. However, it should be noted that this stringent filter could remove potential mutant peptides in samples with low immunopeptidome coverage.

Another challenge is the diverse mutation presentation in the same cancer type across patients and cell lines. In the output from CAN-IMMUNE, we added 1 column in the “metadata” to demonstrate the frequency of the current mutation across cell lines and tissues when generating mutant peptide libraries using all cell lines or tissues. This information will assist users with prioritizing the mutations. Based on these observations, we recommend that users start with a small mutant peptide library for their sample. For example, a cell line-specific library if the cell line is available in CAN-IMMUNE and only expand to all cell lines or all cell lines + tissues when the sample-specific mutation data is unavailable or the number of identified mutant peptides is low. When expanding, we also recommend applying the variant recurrence filter (e.g., variant recurrence = “>=2”) to restrict the library to mutations observed in more than 1 sample of the selected cancer type, which reduces the search space while retaining the most frequently reported mutations. We provided a computational peptide prioritization strategy combining peptide rescoring with Percolator [52], peptide–HLA binding prediction with NetMHCpan 4.2 [44], and optional structural analysis with AlphaFold 3 [57], helping users shortlist high-confidence candidates from the large pool of identified mutant peptides for experimental validation.

Despite these advances, the current version of CAN-IMMUNE has several limitations. First, current mutant peptide libraries are restricted to missense mutations and do not yet encompass other types of mutations [6,7]. Although missense mutations are a major source of cancer neoantigens, frameshift insertions and/or deletions (indels) can also significantly alter the gene transcription and translation, resulting in dysfunctions or pathophysiological processes [70]. In addition, aberrant splicing [71], gene fusions [72], can also contribute potential tumor antigens. In the future, we will incorporate these types of mutations and novel sources of tumor antigens into CAN-IMMUNE. Second, the coverage of certain rare tumor subtypes remains uneven, reflecting biases in the underlying public mutation databases. CAN-IMMUNE is therefore likely most productive for immunogenically hot cancers with high mutational burden; coverage of cold tumors with low mutational burden may be more limited at present, though researchers working on underrepresented cancer types can supplement the database with their own inputs via MutPep. Third, HLA binding was assessed by prediction only. NetMHCpan 4.2 [44] is less accurate for less well-characterized allotypes such as HLA-C and for atypical or noncanonical peptides [73,74] and immunopeptidomes routinely contain predicted nonbinders, including copurified background peptides [8,75,76]. We therefore used predicted binding as only 1 prioritization criterion alongside Percolator probability and Prosit spectral similarity (Fig. 5A) and experimental validation is still required. Future work will focus on expanding the mutation classes supported by CAN-IMMUNE and updating the database as new cancer mutation data become available, with the longer-term goal of coupling the platform with experimental validation workflows to advance neoantigen candidates toward clinical application.

## Conclusions

In this paper, we proposed CAN-IMMUNE, a platform and knowledgebase for LC-MS/MS-based cancer neoantigen discovery. Users can use CAN-IMMUNE to browse, search, and generate bespoke mutant peptide libraries for database search using various search engines. The mutant libraries generated for BC and melanoma enabled the identification of high confidence mutant peptides. Our peptide ranking strategy can prioritize mutant peptides, which can be further experimentally validated. With public availability, CAN-IMMUNE is expected to serve as an effective platform to facilitate the neoantigen discovery, thereby better informing the development of cancer vaccines and immunotherapies.

## Data Availability

The immunopeptidomic raw datasets for breast carcinoma (MDA-MB-231) (RRID:CVCL_0062) and melanoma (LM-MEL-44 and LM-MEL-53) are available via the PRIDE (RRID:SCR_012052) [77] partner repository with dataset identifiers PXD023044 and PXD014397, respectively. The COSMIC database (v104, Cosmic_GenomeScreensMutant_v104_GRCh38.tsv) (RRID:SCR_002260) and CCLE data (DepMap release 26Q1, OmicsSomaticMutations.csv) were used for mutation data curation. All contributing resources with their versions and access dates are listed in Table S1. The BC whole-exome sequencing MAF data was obtained from TCGA GDC (https://portal.gdc.cancer.gov; downloaded 21/03/2025; download link https://portal.gdc.cancer.gov/files/0f463aa5-6351-4c4d-a382-c2f093a34e09; RRID:SCR_014514). The example MAF file and the outputs generated using MutPep are available at https://github.com/sanjaysgk/MutPep/tree/main/data/example. The reference Ensembl human protein translations for GRCh38 (release 112) can also be retrieved using the MutPep command “mutpep-fetch-reference”. CAN-IMMUNE is freely accessible at https://canelib.erc.monash.edu, programmatic access (API) at https://canelib.erc.monash.edu/api/docs, and the source code is available at our GitHub repository (https://github.com/sanjaysgk/CanImmune and https://github.com/sanjaysgk/Mutpep). The complete FragPipe (v24) outputs for all searches reported in this study have been deposited in the Zenodo repository (DOI: 10.5281/zenodo.22803271). These include (a) the peptide-spectrum match, peptide and protein tables, (b) the Percolator input feature files and Percolator output, (c) the CAN-IMMUNE FASTA libraries, and (d) the FragPipe workflow file required to reproduce the searches. We have listed all supplementary data files accompanying this study, with a brief description each in Table S12.

## Supplementary Materials

Supplementary 1 Supplementary Notes Figs. S1 to S13 Tables S1 to S12
