# Supplementary material for: CAN-IMMUNE: A Computational Platform to Facilitate Cancer-Specific Neoantigen Identification
Source: Comput Struct Biotechnol J. 2026 Nov 13;35(1):0274. doi: 10.34133/csbj.0274 (PMC13760139; doi:10.34133/csbj.0274)
Supplement: Supplementary file 1 [file csbj.0274.f1.docx]

**CAN-IMMUNE: a computational platform to facilitate cancer-specific neoantigen identification**

Sanjay S.G. Krishna^1^, Mohammad Shahbazy^1,2^, Kerry A. Mullan^1^, Mohammadreza Dorvash^1,†^, Gabriel Goncalves^1,3,4,5^, Ritchlynn Aranha^1,‡^, Prithvi Raj Munday^1^, Yuheng Jia^6^, Pouya Faridi^1,3,4,5^, Anthony W. Purcell^1,*^, and Chen Li^1,5,*^

^1^Department of Biochemistry and Molecular Biology, Immunity and Cancer Programs, Biomedicine Discovery Institute, Monash University, Clayton, VIC, Australia

^2^Department of Immunobiology, Yale School of Medicine, New Haven, CT, USA

^3^Monash Proteomics & Metabolomics Platform, Department of Biochemistry and Molecular Biology, Monash Biomedicine Discovery Institute, Monash University, Clayton, VIC, Australia

^4^Centre for Cancer Research, Hudson Institute of Medical Research, Clayton, VIC, Australia

^5^Department of Medicine, School of Clinical Sciences, Monash University, Clayton, VIC, Australia

^6^School of Computer Science and Engineering, Southeast University, Nanjing, 210096, Jiangsu, China

^*^To whom correspondence should be addressed: [Chen.Li@monash.edu](mailto:Chen.Li@monash.edu) and [Anthony.Purcell@monash.edu](mailto:Anthony.Purcell@monash.edu)

^†^Current address: Melbourne Mass Spectrometry and Proteomics Facility, Bio21 Molecular Science and Biotechnology Institute, The University of Melbourne, Melbourne, Victoria, Australia.

^‡^Current address: Perlmutter Cancer Center, New York University Grossman School of Medicine, New York, NY 10016, USA.

**Supplementary Tables**

**Table S1. Public resources contributing mutation data to CAN-IMMUNE with publication source PMID is given where a citable publication exists**

| **Resource** | **Role in CAN-IMMUNE** | **Version** | **PMID** |
| --- | --- | --- | --- |
| **Primary mutation sources** | | | |
| COSMIC (1)  https://cancer.sanger.ac.uk/cosmic | Catalogue of Somatic Mutations in Cancer. Supplies the majority of CAN-IMMUNE mutations, from both the Cell Lines Project and patient tumour samples. Provides the COSV genomic variant identifier, transcript accession, CDS and protein change used throughout. | v104 | 30371878 |
| DepMap/CCLE (2)  https://depmap.org | Cancer Dependency Map and Cancer Cell Line Encyclopedia. Supplies somatic mutation calls for cancer cell lines from whole-exome sequencing, together with the ACH model identifier and GRCh38 genomic coordinate. | 26Q1 | 31068700 |
| TSNAdb v2.0 (3) https://pgx.zju.edu.cn/tsnadb | Tumour-specific neoantigen database. SNV-derived predicted neoantigens across 16 tissue types, computed from TCGA. Contributes variants do not present in COSMIC or DepMap; entries carry no per-variant publication because they are computational predictions rather than literature reports. | v2.0 | 36209954 |
| CEDAR (4)  https://cedar.iedb.org | Cancer Epitope Database and Analysis Resource. Literature-curated cancer epitopes; supplies experimentally reported neoantigens with the IEDB epitope identifier and the PubMed ID of the reporting study. | accessed 2026 | 42689115 |
| NEPdb (5) https://nep.whu.edu.cn | Database of T-cell experimentally validated neoantigens; supplies validated neoantigen variants with a resolvable reference record identifier. | accessed 2026 | 33927717 |
| Neodb (6) https://liuxslab.com/Neodb | Comprehensive neoantigen database and discovery platform; supplies curated neoantigen variants with the PubMed ID of the reporting study. | accessed 2026 | 37311149 |
| dbPepNeo2.0 (7) http://www.biostatistics.online/dbPepNeo2 | Database of human tumour neoantigen peptides derived from mass spectrometry and T-cell receptor recognition; supplies MS-observed neoantigen variants. | v2.0 | 35493528 |
| Direct publications (8, 9) | Two studies contribute cell-line mutation calls directly: deep whole-genome sequencing of COLO-829, HCC-1143 and HCC1187; and a signalling-network study of 16 breast cancer cell-line samples. | see refs | 31836783; 24075989 |
| **Reference and annotation resources** | | | |
| UniProt (10) https://www.uniprot.org | Reference protein sequences used to validate the substituted residue and to extract the flanking sequence for each 25-mer. Also supplies the UniProt accession shown on each mutation record. | 2025 release | 39552041 |
| Ensembl https://ensembl.org | Transcript models (ENST accessions). Each mutation is reported against the transcript on which it was called, so isoform-specific coordinates are preserved. | GRCh38 |  |
| GTEx (11) https://gtexportal.org | Gene- and transcript-level tissue expression, shown on each mutation record so candidates can be prioritised by whether the transcript is expressed in the relevant tissue. | v8 | 32913098 |
| Cellosaurus (12) https://www.cellosaurus.org | Cell-line identity, synonyms, CVCL accession and HLA typing. Used to merge cell-line name variants into one canonical entry and to resolve conflicting HLA calls between sources. | accessed 2026 |  |
| Cell Model Passports (13) https://cellmodelpassports.sanger.ac.uk | Cell-line metadata including tissue, cancer type, MSI status and growth properties; underpins the curated cancer-type taxonomy. | accessed 2026 | 30260411 |
| OncoTree (14) https://oncotree.mskcc.org | Cancer-type ontology used for the lineage grouping applied to samples from both cell-line and tissue sources. | accessed 2026 | 33625877 |
| **Prediction resources** | | | |
| NetMHCpan-4.2 (15) | On-demand MHC class I binding prediction for selected HLA alleles, run per mutation record and cached; reports percentile rank and binder classification for the mutant peptide and its wild-type counterpart. | 4.1 | 32406916 |

**Table S2. Breakdown of CAN-IMMUNE v0.3 all data sources**

| **Descriptions** | **Value** |
| --- | --- |
| COSMIC v104 tissue export, records as downloaded | 62,765,128 |
| COSMIC v104 Cell Lines Project export, records | 6,055,938 |
| DepMap 26Q1 OmicsSomaticMutations, records | 1,172,688 |
| DepMap 26Q1 models, as downloaded | 2,154 |
| Retained**: COSMIC tissue | 12,770,178 |
| Retained**: COSMIC Cell Lines Project | 1,229,740 |
| Retained**: DepMap / CCLE | 670,319 |
| Retained**: literature and neoantigen databases | 56,873 |
| **Total mutation records** | **14,727,110** |
| of which missense | 14,621,743 (99.28%) |
| of which complex substitution | 105,367 (0.72%) |
| Unique variants (gene + AA change) | **6,812,413** |
| Unique mutant peptides | **4,531,600** |
| Mutation records with a 25 aa peptide | 14,146,843 (96.06%) |
| Mutation records with a peptide shorter than 25 aa | 580,267 (3.94%) |
| Peptide length, effective range | 13 to 25 |
| Genes | **20,081** |
| Samples, summed across sources | **52,774** |
| Cancer cell lines | **2,052** |
| Patient tumour samples | **49,611** |
| Tissue lineages (OncoTree) | **31** |
| Histology types | **163** |
| Curated cancer types | **55** |

** total number of mutations (not unique) stored in CAN-IMMUNE and the key numbers used in manuscript are shown in bold.

**Table S3. Contribution of each data source to CAN-IMMUNE**

| **Data source** | **#Unique mutant peptides** | **#Unique variants** | **#Genes** | **#Samples** |
| --- | --- | --- | --- | --- |
| COSMIC tissue samples | 3,888,180 | 5,965,729 | 19,082 | 49,685 |
| COSMIC Cell Lines Project | 491,056 | 753,585 | 18,342 | 1,019 |
| DepMap / CCLE | 585,662 | 586,187 | 18,848 | 1,968 |
| Published literature and neoantigen databases | 55,189 | 55,534 | 14,090 | 102 |
| **Total**  **(after redundancy removal)** | **4,531,600** | **6,812,413** | **20,081** | **51,663** |

**Table S4. Samples contributed by each source and composition of the literature-derived source**

**Table S4A. Samples contributed by each source**

| **Data source** | **#Cell lines** | **#Tissue samples** | **Total** |
| --- | --- | --- | --- |
| COSMIC tissue samples | 157 | 49,528 | 49,685 |
| DepMap / CCLE | 1,968 | 0 | 1,968 |
| COSMIC Cell Lines Project | 1,019 | 0 | 1,019 |
| Published literature and neoantigen databases | 19 | 83 | 102 |

**Table S4B. Composition of the literature-derived source**

| **Data source** | **#Mutation records** | **#Unique mutant peptides** | **#Unique variants** | **#Genes** | **#Samples** |
| --- | --- | --- | --- | --- | --- |
| TSNAdb | 41,224 | 41,169 | 41,222 | 11,580 | 16 |
| CEDAR | 10,209 | 10,202 | 10,209 | 6,592 | 24 |
| PubMed | 3,164 | 3,138 | 3,041 | 2,539 | 19 |
| NEPdb | 1,392 | 1,377 | 1,390 | 1,233 | 9 |
| Neodb | 571 | 513 | 560 | 496 | 18 |
| dbPepNeo | 313 | 309 | 313 | 299 | 16 |

**Table S5. Classification levels used in CAN-IMMUNE**

| **Level** | **Count** | **Definition and use** |
| --- | --- | --- |
| Tissue sites of origin | 42 | Anatomical site of the sample. Used by the tissue browse pages. |
| Tissue lineages | 31 | Coarser OncoTree grouping of the same samples; merges sites that the anatomical vocabulary separates (for example large and small intestine). |
| Histology types | 163 | Morphological tumour classification. |
| Curated cancer types | 55 | Two-level harmonised taxonomy used for filtering and recurrence. |
| Genes | 20,081 | Genes carrying at least one neoepitope-producing mutation. |

**Table S6. Fields of the CAN-IMMUNE FASTA header and it’s metadata format information**

| **#** | **Field** | **Example** | **Description** |
| --- | --- | --- | --- |
| 1 | CAN-IMMUNE accession | CI-5210689 | Stable identifier for this mutation record. Resolves at canelib.erc.monash.edu/mutation/CI-5210689. |
| 2 | Gene symbol | NDUFS2 | HGNC gene symbol. |
| 3 | Protein change | p.D462N | HGVS protein-level description, relative to the transcript in field 5. |
| 4 | Sequence-type tag | mutant14mer_D13N | Sequence type, peptide length, and the substitution numbered within the peptide. See below. |
| 5 | Transcript | ENST00000367993.7 | Ensembl transcript the mutation was called on and from which the peptide was extracted. |
| 6 | Source database | cosmic_CL | One of cosmic_TS, cosmic_CL, depMap or pubMed. |
| 7 | Sample | MZ7-mel | Cell line or tumour sample. For the neoantigen databases this field contains colons (for example CEDAR:Lung:Carcinoma), so split headers on the pipe, not the colon. |

The substitution in the tag is numbered within the peptide, not within the protein. In mutant14mer_D13N, D13N means the aspartate at position 13 of the peptide was replaced by asparagine; the same change is p.D462N in protein coordinates. Because the number is peptide-locally relative to the sequence in the FASTA file, it always points to the substituted residue whatever the window length. One expression parses in every tag:

Regex pattern = ^(mutant|wildtype)(\d+)mer(?:_([A-Z])(\d+)([A-Z]))?$

**Table S7. Columns in CSV exports**

| **Column** | **Description** |
| --- | --- |
| CANLIB_ID | CAN-IMMUNE accession, as FASTA field 1. |
| Gene | HGNC gene symbol. |
| Sample | Cell line or tumour sample name. |
| Primary_Site | Tissue site of origin of the sample. |
| Mutation_AA | HGVS protein change, as FASTA field 3. |
| Mutation_CDS | HGVS coding-sequence change, for example c.928G>A. |
| Type | Mutation class: missense substitution or complex substitution. |
| Source | Source database, as FASTA field 6. |
| Transcript | Ensembl transcript accession, as FASTA field 5. |
| Wild_Peptide | Wild-type sequence of the window. |
| Mutant_Peptide | Mutant sequence of the window. |
| 9mer_MutAtPos2 | 9-mer window with the substitution at position 2. |
| 9mer_MutAtPos9 | 9-mer window with the substitution at position 9. |
| Source_ID | Record identifier in the source database: a COSV variant ID for COSMIC, an ACH model ID for DepMap, or a PubMed ID or sub-database key for the literature source. |
| Reference_Link | Resolvable URL for that source record. |

**Table S8. Mutant peptide library generation for Breast cancer and melanoma***

| **Library** | **API query** | **Deposited FASTA file** | **Unique FASTA entries** | **Source records** | **Description** |
| --- | --- | --- | --- | --- | --- |
| MDA-MB-231 | cell_line=MDA-MB-231 project=CL (no recurrence filter) | CANIMMUNE_MDA-MB-231_peptides_unique.fasta | 489 | 1,369 | MDA-MB-231 cell line only. No recurrence filter was applied to this arm, so its counts are not on the same axis as the other four. |
| Breast carcinoma CL | cancer_type_v2=BreastCarcinoma project=CL ct_recurrence_min=2 | CANIMMUNE_BreastCarcinoma_CL_CTrec2_unique.fasta | 12,576 | 35,570 | Breast carcinoma cell lines, including MDA-MB-231. |
| Breast carcinoma CL+TS | cancer_type_v2=BreastCarcinoma ct_recurrence_min=2 | CANIMMUNE_BreastCarcinoma_CL_and_TS_CTrec2_unique.fasta | 25,516 | 116,160 | Breast carcinoma cell lines together with breast carcinoma tissue samples. |
| Melanoma CL | cancer_type_v2=Melanoma project=CL ct_recurrence_min=2 | CANIMMUNE_Melanoma_CL_CTrec2_unique.fasta | 22,800 | 70,861 | Melanoma cell lines |
| Melanoma CL+TS | cancer_type_v2=Melanoma ct_recurrence_min=2 | CANIMMUNE_Melanoma_CL_and_TS_CTrec2_unique.fasta | 110,515 | 579,454 | Melanoma cell lines together with melanoma tissue samples. |

*These files are available at https://github.com/sanjaysgk/CanImmune/tree/new-features/data and are deposited with this submission.<https://github.com/sanjaysgk/CanImmune/tree/new-features/data>

**Table S9. Parameter configuration for searching both TNBC and melanoma immunopeptidomic datasets in FragPipe**

| **Parameter** | **Value** | **Description^+^** |
| --- | --- | --- |
| Precursor mass tolerance | ±20 ppm | Lower and upper bounds of the precursor mass tolerance window |
| Precursor mass units | 1 | Units for precursor mass tolerance (0 = Da, 1 = ppm) |
| Fragment mass tolerance* | ± 20 ppm | Fragment ion mass tolerance window (± this value) |
| Fragment mass units | 1 | Fragment mass tolerance units (0 for Da, 1 for ppm) |
| Calibrate mass | 2 | Perform mass calibration (0 for OFF, 1 for ON, 2 for ON and find optimal parameters) |
| Search enzyme | Nonspecific | Enzymatic cleavage specificity (nonspecific digestion) |
| Variable modifications | M (oxidation) (+15.9949 Da)  NQ (deamidation) (+0.984016 Da)  N-term acetylation (+42.0106 Da) | Post-translational modifications searched |
| Peptide length range | 7-25 amino acids | Minimum and maximum peptide length for database search |
| Fixed modifications | None | Carbamidomethyl (C) is defined in the workflow but not enabled |
| Max variable modifications per peptide | 3 | Maximum number of variable modifications permitted on a single peptide. |
| Max variable modification combinations | 5000 | Maximum number of modified forms enumerated per peptide. |
| Isotope error | 0/1 | Precursor isotope peaks considered (monoisotopic and first isotope). |
| Precursor true tolerance | 20 ppm | Precursor tolerance applied after mass calibration. |
| Mass offsets / open search | 0 (closed search) | No mass offsets applied; delta-mass localisation disabled (localize_delta_mass=false). |
| Missed cleavages | Not applicable | Digestion is nonspecific. The workflow file carries allowed_missed_cleavage=2, but this setting has no effect under nonspecific search. |
| Decoy tag | rev_ | Reversed-sequence decoys and common contaminants were appended by FragPipe. |
| PeptideProphet options | --decoyprobs --ppm --accmass --nonparam --expectscore | Options used for PSM-level probability modelling. |
| FDR filtering | PSM 1%, peptide 1% | Philosopher filter run with --razor --picked --sequential. |
| Protein-level FDR | Not applied | Philosopher filter run with --prot 1, i.e. no protein-level FDR threshold, as is standard for immunopeptidomic data. |
| Group FDR | --razor --picked --sequential --prot 1 --pep 0.01 --psm 0.01 –group with group_variable=2 | FDR estimated separately within protein-evidence (PE) groups; this provides FDR control for the mutant peptide subset. |
| FragPipe / MSFragger / Philosopher versions | FragPipe v24.0, MSFragger v4.4.1, Philosopher v5.1.3-RC9. Rescoring used Percolator v3.07.1 with MSBooster v1.4.14 | Software versions used for the database search. |

*Fragment mass tolerance was set to ±20 ppm, which FragPipe automatically refines during mass calibration (see FragPipe workflow settings [documentation](https://fragpipe.nesvilab.org/docs/tutorial_offset.html)).

^+^parameter definitions are available in the FragPipe workflow settings [files](https://github.com/Nesvilab/FragPipe/blob/develop/workflows/Nonspecific-HLA.workflow).

**Table S10. Mutant peptides identified in TNBC cells using MDA-MB-231-specific mutant peptide library**

| Peptide | IFNg treatment | ENSEMBLE | UniProt | Protein Description | Mutation | Gene | Tumour specific gene description^*^ | Publication | CAN-IMMUNE ID |
| --- | --- | --- | --- | --- | --- | --- | --- | --- | --- |
| AEGRPAPQI | IFNg+ and IFNg- | ENST00000361127.6 | O94898 | Leucine-rich repeats and immunoglobulin-like domains protein 2 | H628R | LRIG2 | LRIG2 regulates cell proliferation, migration and apoptosis of osteosarcoma. The transmembrane protein LRIG2 increases tumour progression in skin carcinogenesis. | [Link](https://bmccancer.biomedcentral.com/articles/10.1186/s12885-022-10123-3) | CI-5743814 |
| AMAKIQAKV | IFNg+ | ENST00000405240.5 | Q9NQC3 | Reticulon-4 | I1184V | RTN4 | RTN4 (Nogo) is aberrantly expressed in multiple carcinomas and contributes to tumour growth and metastasis. Knockout of the RTN4 isoform Nogo-B suppresses hepatocellular carcinoma cell proliferation and tumour growth via the IL-6/STAT3 signalling pathway. | [Link](https://pubmed.ncbi.nlm.nih.gov/28628795/) | CI-5241663 |
| KENSQKIKV | IFNg+ | ENST00000619920.4 | P17980 | 26S proteasome regulatory subunit 6A | E78Q | PSMC3 | Expression of PSMC3 is found in breast cancer. PSMC3 also inhibits prostate cancer cell proliferation by downregulating DGCR8. | [Link](https://www.proteinatlas.org/ENSG00000165916-PSMC3/pathology%20\|%20https:/www.ncbi.nlm.nih.gov/gene/5702#:~:text=Circ%20PSMC3%20inhibits%20prostate%20cancer,182%2D5p%2FNME2%20Axis.) | CI-5170452 |
| KIQAKVPGL | IFNg+ | ENST00000357732.8 | Q9NQC3 | Reticulon-4 | I384V | RTN4 | RTN4 (Nogo) is aberrantly expressed in multiple carcinomas and contributes to tumour growth and metastasis. Knockout of the RTN4 isoform Nogo-B suppresses hepatocellular carcinoma cell proliferation and tumour growth via the IL-6/STAT3 signalling pathway. | [Link](https://pubmed.ncbi.nlm.nih.gov/28628795/) | CI-5675470 |
| LLNPHTLGL | IFNg+ | ENST00000331569.6 | Q9H7S9 | Zinc finger protein 703 | R535L | ZNF703 | ZNF703 is the driver oncogene of the 8p12 amplicon in luminal B breast cancer. Amplification and overexpression activate stem-cell-related gene expression and are associated with poor prognosis and tamoxifen resistance. | [Link](https://pubmed.ncbi.nlm.nih.gov/21337521/) | CI-5713448 |
| MSVEILINV | IFNg+ and IFNg- | ENST00000546190.5 | O60242 | Adhesion G protein-coupled receptor B3 | R624I | ADGRB3 | ADGRB3 (BAI3) is a brain-specific angiogenesis inhibitor of the adhesion GPCR family and a putative tumour suppressor. It is recurrently mutated in lung squamous cell carcinoma and adenocarcinoma and is epigenetically silenced in WNT-subgroup medulloblastoma. | [Link](https://aacrjournals.org/cancerres/article/78/19_Supplement/B17/631653/Abstract-B17-ADGRB3-is-a-novel-tumor-suppressor) | CI-20901082 |
| QEVEQGVKFSL | IFNg+ | ENST00000299705.10 | Q9Y3Q3 | Transmembrane emp24 domain-containing protein 3 | E43Q | TMED3 | TMED3 behaves as an oncogene that promotes the proliferation and migration of breast cancer cells by activating Wnt/β-catenin signalling. Strategies targeting TMED3 have potential therapeutic implications for patients with breast cancer. | [Link](https://www.ncbi.nlm.nih.gov/pmc/articles/PMC7311187/#:~:text=for%20poor%20prognosis.-,TMED3%20behaves%20as%20an%20oncogene%20that%20promotes%20the%20proliferation%20and,for%20patients%20with%20breast%20cancer.) | CI-19996288 |
| TEVSESIKA | IFNg- | ENST00000372979.9 | O95905 | Protein ecdysoneless homolog | M457I | ECD | ECD (ecdysoneless) promotes cell-cycle progression by relieving Rb-family repression of E2F. It is overexpressed in breast cancer, where high expression correlates with higher histological grade and shorter survival in HER2/neu-overexpressing patients. | [Link](https://pubmed.ncbi.nlm.nih.gov/22270930/) | CI-19976390 |

*The functional description of the tumour specific genes was extracted from the papers listed in the ‘Publication’ column.

**Table S11. Functional comparison of CAN-IMMUNE with existing mutant peptide and neoantigen databases**

| **Feature** | **CAN-IMMUNE (v0.3)** | **XMAn v2** | **CusVarDB** | **Neodb** | **dbPepNeo2.0** | **TSNAdb v2.0** | **NEPdb** | **CEDAR** |
| --- | --- | --- | --- | --- | --- | --- | --- | --- |
| **Primary content** | Cancer-specific mutant peptide libraries | Mutated peptide FASTA | Sample-specific variant database builder | Validated and predicted neoantigens | MS/immunoassay-validated neoantigen peptides | Predicted and validated neoantigens | Validated neoantigens and ineffective neoepitopes | Curated cancer epitope and immune receptor (TCR/BCR) records |
| **Data scale** | 6,812,413 unique variants; 4,531,600 unique mutant peptides | 2,539,031 non-redundant entries (2,377,103 missense; 161,928 nonsense) from 17,599 proteins | Not a fixed database user-generated | Largest reported collection of experimentally validated neoantigens | 801 high-confidence; 842,289 low-confidence immunopeptidomes | 372,273 SNV, 137,130 INDEL, 11,093 fusion-derived; 1,856 validated | >17,000 validated immunogenic and non-immunogenic neoepitopes | 224,355 cancer-associated epitopes, including >16,100 neoepitopes (31 July 2022 release) |
| **Mutation source** | COSMIC v104 (GRCh38, Feb 2026), DepMap 26Q1, literature and curated neoantigen databases | COSMIC V88 | User-provided NGS data | Literature and recurrent driver mutations | Literature and public MS datasets | TCGA | Literature and TCGA | Published literature (manual curation) |
| **Mutation types** | Missense (SNV) | Missense and nonsense | Variant-dependent | SNV/INDEL-derived | Not specified | SNV, INDEL, gene fusion | SNV-derived | Not mutation-centric; epitope-level (neoantigen, viral, self-antigens) |
| **Cell line-level resolution** | Yes (2,052 cell lines) | No | User sample only | No | No | No | No | No |
| **Tissue / histology-level resolution** | Yes (42 tissue sites of origin, 31 OncoTree lineages, 163 histology types, 55 curated cancer types) | No | No | Partial | Tumour type only | Tissue and gene-specific | Cancer type annotated | Cancer type annotated |
| **Bespoke library generation (user-defined subset)** | Yes (Browse/Search with download) | No (single comined samples FASTA) | Yes (from user NGS data) | No | No | No | No | No |
| **Programmatic access (REST API)** | Yes | No | No | No | No | No | No | Yes |
| **Local/offline library generation from user data** | Yes (MutPep; VCF/MAF/COSMIC/custom input) | No | Yes | No | No | No | No | No |
| **Ready-to-use format for MS search engines** | Yes (FASTA; FragPipe, DIA-NN, PEAKS) | Yes (FASTA) | Yes (FASTA) | No | No | No | No | No |
| **Mutation frequency metadata across samples** | Yes | No | No | No | No | Shared-neoantigen analysis | No | No |
| **Peptide prioritisation workflow** | Yes (PeptideProphet, NetMHCpan 4.1, PANDORA, AlphaFold2) | No | No | Yes (prediction tools and Immuno-GNN) | Yes (DeepCNN-Ineo, BLASTdb) | Yes (three-tool consensus and expression filter) | Yes (pan-cancer neoepitope prediction) | Yes (IEDB-style epitope prediction and analysis tools) |
| **Intended application** | LC-MS/MS database search for neoantigen discovery | MS variant peptide search | MS variant peptide search | Immunogenicity prediction and lookup | Neoantigen lookup | Neoantigen lookup | Neoantigen lookup and immunogenicity benchmarking | Epitope and receptor lookup; benchmarking datasets |

MS, mass spectrometry; SNV, single nucleotide variant; INDEL, insertion/deletion; NGS, next-generation sequencing; COSMIC, Catalogue of Somatic Mutations in Cancer; TCGA, The Cancer Genome Atlas; TCR, T cell receptor; BCR, B cell receptor. Data scale figures are as reported in the original publications; databases were accessed on [17/09/2026].

**Table S12. Descriptions of supplementary data files^*^**

| #Id | File name | Description |
| --- | --- | --- |
| 001 | Melanoma_all_predictions_V2_03092026.csv | Mutant peptides identified in LM-MEL-44 and LM-MEL-53 with the two melanoma libraries, with NetMHCpan 4.2 predictions against every HLA class I allotype of each cell line (97 unique peptides, 1,368 peptide-allotype rows). |
| 002 | TNBC_all_predictions_V2_03092026.csv | Mutant peptides identified in MDA-MB-231 with the three breast cancer libraries, with NetMHCpan 4.2 predictions against the six HLA class I allotypes of this cell line (92 unique peptides, 891 peptide-allotype rows). |
| 003 | structural_models_AlphaFold3_V2_03092026.csv | Per-model AlphaFold 3 metrics for the peptide-HLA complexes modelled in this study (21 complexes, 5 models each): ipTM, peptide pLDDT, anchor pLDDT, C-terminal anchor distance, anchor contacts and clash flag. |
| 004 | final_ranked_peptides_list_V2_03092026.csv | Mutant peptides identified in MDA-MB-231 IFNg+ with the breast carcinoma cell lines + tissues library, ranked by the combined score shown in Figure 5A. Gives each of the four measures as recorded and as min-max scaled to 0–25 across this peptide set (predicted binding as −log10 %Rank_EL, differential agretopicity as DAI log2, identification confidence as the Percolator probability and spectral match quality as the MSFragger Hyperscore), their unweighted sum out of 100, the presenting allotype, the wild-type counterpart and its predicted binding (58 peptides. |
| 005 | Figure1F_cancertype_tissue_mutation_distribution_V2_03092026.csv | Mutation records, unique mutant peptides, variants, genes and samples for each of the 55 curated cancer types and 31 tissue lineages, the data plotted in Figure 1F |

*These files are available at <https://github.com/sanjaysgk/CanImmune/tree/new-features/data>.

**Supplementary Figures**

**a**

**b**

**Figure S1. Cross-source overlap of the data in CAN-IMMUNE.** (a) Unique mutant peptides. (b) Unique variants (distinct combination of gene and protein-level amino acid change). Horizontal bars at left give the total from each source. Filled dots in the matrix mark the exact set of sources reporting each group: a single dot means the entries came from that source only, example 322,992 unique variant from COSMIC cell line and connected dots mean they were reported by each source in the set (overlap across source). The groups are therefore mutually exclusive and sum to the total number of distinct entries (4,531,600 peptides in (a), 6,812,413 variants in (b)), while the per-source totals at left sum to more because an entry shared between sources is counted once in each. Vertical bars use a logarithmic axis, as the largest group exceeds the smallest by around five orders of magnitude. Per-source totals are also given in **Supplementary Table S3**.

**Figure S2. Sequence overlaps between the CAN-IMMUNE mutant peptide libraries**. Unique mutation peptides sequence from FASTA. (a) The three breast cancer libraries: MDA-MB-231-specific (489 peptides), all breast cancer cell lines (12,576 peptides) and breast cancer cell lines + tissue samples (25,516 peptides). (b) The two melanoma libraries: all melanoma cell lines (22,800 peptides) and melanoma cell lines + tissue samples (110,515 peptides). Overall, all mutant peptides in cell lines are included in cell line + tissue sample.

**Figure S3. Mutant peptides identified in MDA-MB-231 against different library sizes.** Three CAN-IMMUNE libraries of increasing size were each appended to the same canonical proteome: MDA-MB-231-specific (7 in IFNg+ and 3 in IFNg-), all breast cancer cell lines (24 in IFNg+ and 23 in IFNg-), and breast cancer cell lines + BC tissues (62 in IFNg+ and 37 in IFNg-). Counts are unique peptides at 1% FDR spanning the substituted residue. (a) Identifications increased from 7 to 62 with IFNg+ and from 3 to 37 IFNg-. (b) Where each mutant peptide came from, against a canonical-only search of the same raw spectra: not identified in UniProt (green), previously assigned to its own wild type (blue), or to an unrelated peptide (grey). Most of the gain is the blue group, 38 of 62 with IFNg+ and 28 of 37 IFNg-. (c) Hyperscore of the mutant minus its UniProt wild type, for the blue group. 199 of 214 are exact ties (93%), meaning that in most cases the mutant and the UniProt canonical peptide it replaced matched the spectrum equally well.

**Figure S4. Mutant peptides identified in melanoma cell lines against different library sizes.** Two CAN-IMMUNE libraries were each appended to the same canonical proteome: all melanoma cell lines and melanoma cell lines + skin tissues. Counts are unique peptides at 1% FDR spanning the substituted residue. (a) Identifications increased with library size in three of the four searches: LM-MEL-44 from 21 to 54 with IFNg+, and LM-MEL-53 from 18 to 30 with IFNg+ and 13 to 26 with IFNg-. In LM-MEL-44 IFNg- the seven peptides found with the cell line library were all lost with the larger library (**Figure S10**). (b) Where each mutant peptide came from, against a canonical-only search of the same raw spectra: not identified in UniProt (green), previously assigned to its own wild type (blue), or to an unrelated peptide (grey). No bar is shown for LM-MEL-44 IFNg- with the larger library, where no peptides was identified. Most of the gain is the blue group, 42 of 54 in LM-MEL-44 IFNg+ and 18 of 30 in LM-MEL-53 IFNg+. (c) Hyperscore of the mutant minus its UniProt wild type, for the blue group. 179 of 189 are exact ties (95%), meaning that in most cases the mutant and the UniProt canonical peptide it replaced matched the spectrum equally well.

 **Figure S5. Distribution of peptide-spectrum match probabilities for mutant peptides identified in MDA-MB-231.** Histogram (teal bars) and kernel density estimate (KDE [kernel density estimation], orange curve) of Percolator posterior probabilities across N = 92 unique mutant peptides, pooled from database searches against all three CAN-IMMUNE libraries (MDA-MB-231-specific, breast carcinoma cell lines, and breast carcinoma cell lines + BC tissues), after applying the strict mutant rule. The distribution shows a mean probability of 0.995 (blue dashed line) and a median of 0.996 (pink dotted line). All 92 peptides exceeded 0.9 with the lowest being 0.9852, so no cutoff line was drawn and the axis is shown over 0.98-1.0. The distribution is left-skewed (skewness −0.52), with peak density at probability = 1.0, indicating that the retained identifications are of uniformly high confidence.

**Figure S6. Peptide length distributions and HLA-I binding motifs for MDA-MB-231** across all four search databases (blocks 1 to 4): the canonical proteome (UniProt only), and the canonical proteome extended with the MDA-MB-231-specific, all breast cancer cell lines, and breast cancer cell lines + BC tissues using CAN-IMMUNE. Within each block, rows are the IFNg-untreated (top) and IFNg-treated (bottom) conditions, (a, d) length distribution of the identified peptides as a percentage of the peptides in each class, canonical in grey and mutant in red, with n the number of distinct sequences, (b, e) sequence logo of the canonical 9-mers, (c, f) sequence logo of the mutant 9-mers, or shown ‘n= ’ where fewer than ten mutant 9-mers were identified. Logos are Kullback-Leibler information content in bits, positions 1 to 9 running N to C terminus. The canonical distributions are essentially unchanged across the four databases, showing that extending the search space with a mutant library does not distort the bulk immunopeptidome. Peptides are the distinct sequences identified at 1% FDR with contaminants removed, mutant peptides span the substituted residue and exclude isobaric I/L substitutions. Sequence logos were generated using Logomaker (16).

**Figure S7. Peptide length distributions and HLA-I binding motifs for LM-MEL-44** across all three search databases (blocks 1 to 3): the canonical proteome (UniProt only), and the canonical proteome extended with the all-melanoma cell lines and melanoma cell lines + skin tissues built using CAN-IMMUNE. Panels, colours and definitions are as in **Figure S6** above. In the LM-MEL-44 IFNg+ cell lines + skin tissues peptides library search (block 3 panel c) no mutant peptides identified.

**Figure S8. Peptide length distributions and HLA-I binding motifs for LM-MEL-53** across all three search databases (blocks 1 to 3). Panels, colours and definitions are as in **Figure S6** and **Figure S7** above.

 **Figure S9. Distribution of predicted %Rank_EL across the six HLA class I allotypes of MDA-MB-231 across all breast cancer mutant peptide libraries**. The six allotypes are HLA-A*02:01, HLA-A*02:17, HLA-B*40:02, HLA-B*41:01, HLA-C*02:02 and HLA-C*17:01. Predictions (using netMHCpan-4.2 (15)) were performed using 85 unique 8-14mers (out of 92 peptides in total). Each violin shows the density of %Rank_EL for the full set of 85 peptides against that allotype on a logarithmic axis, with boxes indicating interquartile ranges and white points the medians. figures above shows each violin are the number of peptides reaching %Rank_EL <= 0.5 (strong binder, pink dotted line) and <= 2 (binder, grey dotted line): 21, 23, 12, 10, 8 and 15 strong binders, and 32, 37, 21, 17, 23 and 32 binders, for A*02:01, A*02:17, B*40:02, B*41:01, C*02:02 and C*17:01 respectively. Together, 65 of the 92 peptides are predicted to bind at least one of the six allotypes and 45 to bind at least one strongly, with the HLA-A*02:01and HLA-C*17:01 allotypes accounting for the largest share.

**Figure S10. Mutant peptides retained, lost and gained with library expansion.** (a, b) Mutant peptide sequences identified with the cell line library, split into those identified again with the cell lines + tissues library (retained) and those that were not (lost), together with the sequences identified only with the larger library (gained). Each lost peptide was present in the larger library, so its loss cannot be attributed to absence from the search space. Note that one peptide can be supported by multiple spectra. (c) −log10 E-value of the peptide-spectrum matches (PSMs) supporting each group, taken from the search that identified the peptide (i.e., the cell line library search for retained and lost peptides, and the cell lines + tissues library search for gained peptides). The E-value, reported by MSFragger, is the number of random matches expected to score at least as well as the observed PSM, estimated from the score distribution of all candidate peptides for that spectrum. Higher −log10 E-values indicate more significant matches, and values below 0 correspond to an E-value above 1 (i.e., a match no better than expected by chance). Box plots give the median and interquartile range, and whiskers extend to 1.5x the interquartile range. Points are individual PSMs. Lost peptides were marginal identifications in the smaller search (median −log10 E-value −0.1 against 2.7 for retained peptides in MDA-MB-231, −0.2 against 2.2 in LM-MEL-44, and 1.3 against 3.4 in LM-MEL-53). Across all cell lines, none of the 40 PSMs supporting the lost peptides was reassigned to a different peptide in the larger search. All identifications were at 1% FDR, restricted to peptides spanning the substituted residue, with contaminants removed and isobaric I/L substitutions excluded.

**Figure S11. Overlap of annotated spectra, PSMs and mutant PSMs across MDA-MB-231 searches.** The same raw files were searched against the canonical proteome (UniProt only) and against the canonical proteome extended with each of the three CAN-IMMUNE libraries: MDA-MB-231-specific, breast cancer cell lines, and breast cancer cell lines + BC tissue samples. (a) Spectra annotated at 1% FDR. (b) PSMs, where a spectrum counts as shared only if it was assigned the same peptide. (c) Mutant PSMs, restricted to identifications on a CAN-IMMUNE entry where the peptide spans the substituted residue. In each panel the vertical bars give the number of entries reported by exactly the set of searches marked with filled dots below, so the groups are mutually exclusive and sum to the distinct total (304,093 in (a), 304,642 in (b), 269 in (c)). Orange marks entries found by a single search. Totals for each search are given beside the row labels. A logarithmic axis is used because the largest group exceeds the smallest by more than two orders of magnitude. The canonical proteome shared by all four searches dominates panels (a) and (b). In panel c the canonical-only search yields no mutant PSMs by construction, and mutant PSMs rise with library size: 13, 86 and 241 for the MDA-MB-231, cell line, and cell line + tissue libraries. Spectra of which identification changes between searches are characterised in **Figure S3**. Isobaric I/L substitutions are excluded from panel (c).

**Figure S12. Overlap of annotated spectra, PSMs and mutant PSMs across the three melanoma searches.** Data from LM-MEL-44 and LM-MEL-53 were pooled and searched against the canonical proteome alone (UniProt only) and against the canonical proteome extended with the melanoma cell line library and the melanoma cell lines + tissue samples library from CAN-IMMUNE. Panels, colours and set definitions are as in **Figure S11**. Groups are mutually exclusive and sum to 492,923 annotated spectra in (a), 494,484 PSMs in (b) and 374 mutant PSMs in (c). Panel (b) exceeds panel a because a spectrum assigned a different peptide in different searches contributes more than one PSM. In panel (c), of the 374 mutant PSMs, 60 were found only with the cell line library, 220 only with the cell lines + tissue samples library, and 94 with both.

**Figure** **S13. Comparison of the experimental spectrum for AMAKIQAKV with its Prosit-predicted spectrum.** The upper panel shows the experimental spectrum from the MDA-MB-231 immunopeptidome and the lower panel shows the Prosit-predicted spectrum. Annotated fragment ions are coloured by series (b-ions teal, y-ions orange), with the doubly charged precursor [M+2H]²⁺ marked in grey. Plots generated with the Universal Spectrum Explorer (17).

**Supplementary Notes**

**Supplementary Note S1:**

From the COSMIC Cell Lines Project, we used the ‘Genome Screens Mutant export’, comprising 6,055,938 mutation records across 1,019 cancer cell lines from 30 primary tissue sites and 32 histological classifications. Out of these mutations, 1,339,742 (22.1%) are annotated as missense mutations. On the other hand, the export of patient tumour samples from COSMIC (i.e., tissue samples) comprises 62,765,128 records across 51,887 samples as downloaded, of which 14,683,692 (23.4%) are missense mutations (**Table S2**). Of these, 49,685 samples contribute to CAN-IMMUNE after filtering (**Tables S3** and **S4**). These are somatic mutations curated by COSMIC from cancer samples (18). From DepMap/CCLE, we used the ‘OmicsSomaticMutations’ table of release 26Q1, consisting of 1,172,688 mutation records across 1,968 cancer models of which 965,352 records (82.3%) are missense (**Table S2**). These are somatic mutations identified by sequencing of patient-derived cancer cell lines and other cancer models (2). From published literature (8, 9) and curated neoantigen databases we assembled a further 56,873 records across 102 samples. Full class compositions are given in **Table S2**. Number of mutations, cell lines, and tissues from various resources are listed in **Tables S3** and **S4**. These mutations from various resources were reported per sample and per transcript, Records were filtered to retain only alterations capable of producing a mutant peptide, missense substitutions and complex substitutions resolving to a single amino acid change were retained, while synonymous, nonsense, frameshift, insertion, deletion, splice-site, intronic and untranslated-region variants were excluded. Exact duplicate records were removed and for DepMap, where the export carries multiple sequencing entries for some models, records were resolved to unique variants per model. No further biological filtering was applied, as CAN-IMMUNE is designed to provide comprehensive coverage for users to apply sample, tissue, cancer-type or gene-level filters at the library-generation stage using the metadata fields provided.

**Supplementary Note S2:**

Here are several examples of mutant peptides and their 9 mer forms for prediction from CAN-IMMUNE FASTA. All entries were taken directly from the export API. Each sequence appears on the line below its header.

Example 1: mutant 25-mer peptide and its wild-type form (V🡪I):

>CI-5200838|NDUFS2|p.V310I|mutant25mer_V13I|ENST00000367993.7|cosmic_CL|CHP-134
IQWDLRKTQPYDIYDQVEFDVPVGS
>CI-5200838|NDUFS2|p.V310I|wildtype25mer_V13I|ENST00000367993.7|cosmic_CL|CHP-134
IQWDLRKTQPYDVYDQVEFDVPVGS

Example 2: two 9-mer windows of the same variant, with the substituted residue at position 2 and at position 9 (the main HLA class I anchor positions), respectively. Both come from the mutant peptide, so both are tagged mutant.

>CI-5200838|NDUFS2|p.V310I|mutant9mer_V2I|ENST00000367993.7|cosmic_CL|CHP-134
DIYDQVEFD
>CI-5200838|NDUFS2|p.V310I|mutant9mer_V9I|ENST00000367993.7|cosmic_CL|CHP-134
LRKTQPYDI

Example 3: the mutation occurs at the second position from the C-terminus of the protein. The window was therefore cut short to 14 residues. In this case, for prediction, only one 9-mer window (where the mutation is at position 9) is possible.

>CI-5210689|NDUFS2|p.D462N|mutant14mer_D13N|ENST00000367993.7|cosmic_CL|MZ7-mel
IIGTQDIVFGEVNR
>CI-5210689|NDUFS2|p.D462N|mutant9mer_D9N|ENST00000367993.7|cosmic_CL|MZ7-mel
QDIVFGEVN

Example 4: mutant peptides are from the other sources. Field 6 identifies the source and field 7 the sample; for the aggregated neoantigen databases the sample field encodes sub-database, tissue and histology.

>CI-20063529|RTN4|p.I1184V|mutant21mer_I13V|ENST00000337526.11|depMap|MDA-MB-231
NVKDAMAKIQAKVPGLKRKAE
>CI-20962276|BRAF|p.L674Q|mutant25mer_L13Q|ENST00000496384.7|pubMed|CEDAR:Lung:Carcinoma
RDQIIFMVGRGYQSPDLSKVRSNCP

Example 5: a collapsed entry from a library exported with ‘unique=TRUE’, where one record is emitted per distinct sequence rather than per sample. This option is set to ‘unique’ by default when using CAN-IMMUNE website to mutant peptide library generation and is configurable when using our REST API.

>CI-5164437|TP53|p.G107R|mutant25mer_G13R|ENST00000610623.4|cosmic_CL|HO-1-N-1 n=20 samples=5 also=CI-20013786:NCO2,CI-20013787:IS076A,CI-20013788:IS076P,CI-20506222:HO-1-N-1

When this option is selection, if a mutant peptide (including the mutation and the protein) can be from multiple samples, sources, or transcripts, all the information is summarised in the header. Full field definitions are given in the CAN-IMMUNE API documentation (<https://canelib.erc.monash.edu/api/docs>).

**References**

1.	Wang F, Zhang Z, Mao M, Yang Y, Xu P, Lu S. COSMIC-based mutation database enhances identification efficiency of HLA-I immunopeptidome. J Transl Med. 2024;22(1):144.

2.	Ghandi M, Huang FW, Jane-Valbuena J, Kryukov GV, Lo CC, McDonald ER, 3rd, et al. Next-generation characterization of the Cancer Cell Line Encyclopedia. Nature. 2019;569(7757):503-8.

3.	Wu J, Chen W, Zhou Y, Chi Y, Hua X, Wu J, et al. TSNAdb v2.0: The Updated Version of Tumor-specific Neoantigen Database. Genomics Proteomics Bioinformatics. 2023;21(2):259-66.

4.	Kosaloglu-Yalcin Z, Blazeska N, Vita R, Carter H, Nielsen M, Schoenberger S, et al. The Cancer Epitope Database and Analysis Resource (CEDAR). Nucleic Acids Res. 2023;51(D1):D845-D52.

5.	Xia J, Bai P, Fan W, Li Q, Li Y, Wang D, et al. NEPdb: A Database of T-Cell Experimentally-Validated Neoantigens and Pan-Cancer Predicted Neoepitopes for Cancer Immunotherapy. Front Immunol. 2021;12:644637.

6.	Wu T, Chen J, Diao K, Wang G, Wang J, Yao H, et al. Neodb: a comprehensive neoantigen database and discovery platform for cancer immunotherapy. Database (Oxford). 2023;2023.

7.	Lu M, Xu L, Jian X, Tan X, Zhao J, Liu Z, et al. dbPepNeo2.0: A Database for Human Tumor Neoantigen Peptides From Mass Spectrometry and TCR Recognition. Front Immunol. 2022;13:855976.

8.	Arora K, Shah M, Johnson M, Sanghvi R, Shelton J, Nagulapalli K, et al. Deep whole-genome sequencing of 3 cancer cell lines on 2 sequencing platforms. Sci Rep. 2019;9(1):19123.

9.	Zaman N, Li L, Jaramillo ML, Sun Z, Tibiche C, Banville M, et al. Signaling network assessment of mutations and copy number variations predict breast cancer subtype-specific drug targets. Cell Rep. 2013;5(1):216-23.

10.	UniProt C. UniProt: the Universal Protein Knowledgebase in 2023. Nucleic Acids Res. 2023;51(D1):D523-D31.

11.	Consortium GT. The GTEx Consortium atlas of genetic regulatory effects across human tissues. Science. 2020;369(6509):1318-30.

12.	Bairoch A. The Cellosaurus, a Cell-Line Knowledge Resource. J Biomol Tech. 2018;29(2):25-38.

13.	van der Meer D, Barthorpe S, Yang W, Lightfoot H, Hall C, Gilbert J, et al. Cell Model Passports-a hub for clinical, genetic and functional datasets of preclinical cancer models. Nucleic Acids Res. 2019;47(D1):D923-D9.

14.	Kundra R, Zhang H, Sheridan R, Sirintrapun SJ, Wang A, Ochoa A, et al. OncoTree: A Cancer Classification System for Precision Oncology. JCO Clin Cancer Inform. 2021;5:221-30.

15.	Nilsson JB, Greenbaum J, Peters B, Nielsen M. NetMHCpan-4.2: improved prediction of CD8+ epitopes by use of transfer learning and structural features. Front Immunol. 2025;16:1616113.

16.	Tareen A, Kinney JB. Logomaker: beautiful sequence logos in Python. Bioinformatics. 2020;36(7):2272-4.

17.	Schmidt T, Samaras P, Dorfer V, Panse C, Kockmann T, Bichmann L, et al. Universal Spectrum Explorer: A Standalone (Web-)Application for Cross-Resource Spectrum Comparison. J Proteome Res. 2021;20(6):3388-94.

18.	Sondka Z, Dhir NB, Carvalho-Silva D, Jupe S, Madhumita, McLaren K, et al. COSMIC: a curated database of somatic variants and clinical data for cancer. Nucleic Acids Res. 2024;52(D1):D1210-D7.
